# New insights into the first cervical vertebrae of *Otavipithecus* and *Nacholapithecus*

**DOI:** 10.1038/s41598-025-09006-x

**Published:** 2025-07-08

**Authors:** Amélie Beaudet, Yasuhiro Kikuchi, Fredrick Kyalo Manthi, Emmanuel Ndiema, Dominic Stratford, Bernhard Zipfel

**Affiliations:** 1 Laboratoire de Paléontologie, Évolution, Paléoécosystèmes et Paléoprimatologie (PALEVOPRIM), UMR 7262 CNRS & Université de Poitiers, 6 Rue Michel Brunet, Poitiers, 86000 France; 2 Department of Archaeology, University of Cambridge, Henry Wellcome Building, Fitzwilliam St, CB2 1QH Cambridge, England; 3 School of Geography, Archaeology and Environmental Studies, University of the Witwatersrand, Private Bag 3, Johannesburg, 2050 South Africa; 4 Division of Human Anatomy and Biological Anthropology, Department of Anatomy and Physiology, Faculty of Medicine, Saga University, Saga, 849- 8501 Japan; 5 National Museums of Kenya, Kipande Road, Nairobi, Kenya; 6 Department of Anthropology, Stony Brook University, Stony Brook, NY 11794-4364 USA; 7 Evolutionary Studies Institute, University of the Witwatersrand, Private Bag 3, WITS Johannesburg, 2050 South Africa

**Keywords:** Atlas, Afropithecidae, Locomotion, Posture, Geometric morphometrics, Palaeontology, Bone

## Abstract

Fossil hominoids are crucial to understand the selection pressures that played a role in the emergence of modern hominoid positional behaviors. Here we investigate the morphology of the atlas of *Otavipithecus namibiensis* (GSN BA 104’91, Namibia) and *Nacholapithecus kerioi* (KNM-BG 35250BE, Kenya) for identifying potential positional-related signals and discussing functional and evolutionary implications. Published data from GSN BA 13’21, a second *Otavipithecus* atlas from Namibia, were integrated. For comparative material, 105 atlases of extant catarrhines and platyrrhines were included. In addition to standard linear measurements, the morphology of GSN BA 104’91 and KNM-BG 35250BE was investigated by landmark-based geometric morphometric (GM) method and statistical analyses. The dimensions of the Miocene specimens fall within, or closely approximate to, the range of variation of *Pan* and *Hylobates*. Our GM analyses indicate that GSN BA 104’91 is more similar to *Pan*. When the right lateral mass only is considered, GSN BA 104’91 and KNM-BG 35250BE show similarities with hominoids and cercopithecoids. Our results possibly support a positional repertoire in *Otavipithecus* that would have been partly similar with extant hominoids, and in particular with *Pan* (e.g., terrestrial quadrupedalism, climbing), and the presence of a mix of hominoid-like and cercopithecoid-like traits in the axial skeleton of *Nacholapithecus*.

**Supplementary Information:**

The online version contains supplementary material available at 10.1038/s41598-025-09006-x.

## Introduction

The evolutionary context of the emergence of hominin bipedalism, as well as the nature of the ancestral model for the *Pan*-*Homo* last common ancestor, remain largely questioned^1^. The orthograde body plan shared by all extant hominoids has been debated as one of the key characters that could have been potentially inherited from a common ancestor and co-opted for habitual bipedalism in hominins^1,2^. As such, identifying and clarifying the nature and polarity of position-related traits in the skeleton of the ancestors and fossil distant relatives of the hominins has the potential to shed new light on this question^3^. In particular, due to its role in overall trunk stability and mobility, as well as in posture and locomotion, the vertebral column represents a region of interest for reconstructing extant and fossil primate positional behaviors^4–6^ including fossil hominoids^7–10^.

Besides acting as the interface between the head and the axial skeleton, the first cervical vertebra (atlas) is involved in the mechanisms that direct and stabilize head movements^11,12^. Variation in the morphology of the atlas, particularly in the configuration and orientation of the arches and superior articular facets, has been proven to be correlated with positional behaviours in extant primates^13–15^. As such, Manfreda et al. (2006)^13^ identified a series of traits in the primate atlas that discriminates orthograde species from pronograde species, the former being characterized by thin arches, more posteriorly and inferiorly oriented transverse processes and more inclined and laterally rounded superior articular facets. Similarly, Nalley & Grider-Potter (2017)^12^ demonstrated that the relative posterior arch length is correlated with neck posture, and that the superior facet curvature is greater in primates with more horizontal neck. In addition to skeletal variation, the architecture of the ligaments and the muscular anatomy of the atlas are functionally informative, in particular since the atlas provides attachment for upper limb musculature and soft tissues involved in head stability and mobility^11^. Although the role and prevalence of some of these structures remain enigmatic (e.g., absence of nuchal ligament in great apes, presence of atlanto-clavicularis muscle in apes^11,12,16^), their influence on the morphology of the atlas (e.g., tubercles) can be used as a proxy to reconstruct the movement repertoire of the neck and elaborate functional hypotheses, as well as to identify phylogenetic signals. For example, Nalley & Grider-Potter (2019)^12^ hypothesized that the cervical vertebrae of primate with horizontal neck postures should show increased length of the transverse and spinous processes and larger cross-sectional areas of the neural arch for increasing mechanical advantage of the muscles and resistance to bending loads. Additionally, Gómez-Olivencia et al. (2007)^17^ proposed that the degree of development of tubercles, which serve as sites for attachment of the transverse ligament of the atlas, could have phylogenetic significance within hominins and may have direct applications for the study of fossil hominins^18^.

Although tracking evolutionary changes in the axial skeleton is a challenging task due to the fragmentary nature of the fossil record^19^, first cervical vertebrae are frequently recovered from fossil hominoid-bearing sites, including atlases of Miocene apes from southern and eastern Africa^10,20–22^. A virtually complete atlas was discovered from the breccia block #BA 91–104 in the locality of Berg Aukas, northern Namibia, and attributed to the Miocene hominoid species *Otavipithecus*^20^. The overall dimensions were suggested to approximate those of a female *Papio ursinus*^20^. The morphology of GSN BA 104’91 was described as presenting a mix of quadrupedal cercopithecoid-like (e.g., horizontal orientation of the articular facets, reduced transverse processes) and orthograde hominoids (e.g., narrow anterior arch supero-inferiorly) or intermediate (e.g., articular facets less steeply inclined in the transverse plane than in cercopithecoids but more than in hominoids) features^20,23^. A second partial atlas from the same locality has been recently published and assigned to the same species^24^. GSN BA 13’21 is smaller than GSN BA 104’91 but shares similar features with hominoids and GSN BA 104’91 (e.g., horizontal orientation of the articular facets, reduced transverse processes)^24^. *Otavipithecus* is a medium-sized middle Miocene hominoid (14–20 kg) from Namibia dated to 12–13 million years ago (Ma) by biochronology that represents, together with remains identified as cf. *Kenyapithecus* sp., the southernmost evidence of Miocene apes to date^20,25^. The phylogenetic position of *Otavipithecus* is uncertain, but cladistic analyses indicate affinities with *Afropithecus* (sister group to *Kenyapithecus* and extant apes^26^). The postcranial remains of *Otavipithecus* indicate a repertoire with an arboreal component, likely quadrupedalism^23,24^.

In 1996–1998, a partial hominoid skeleton preserving an incomplete atlas (KNM-BG 35250BE) was unearthed in the site BG-K in Nachola, northern Kenya, and ascribed to a newly erected genus and species, *Nacholapithecus kerioi*^27^. *Nacholapithecus* is a large-bodied middle Miocene hominoid (with a range of 20–23 kg estimated using males only) found in Kenya and dated to 15 million years old^27^. *Nacholapithecus*, classified within the subfamily of the equatorines, is considered as a possible stem hominoid^28^. The comparative anatomy and proportions of the skeleton of *Nacholapithecus* suggests arboreal quadrupedalism as the main positional behavior combined with upright posture and occasional antipronograde locomotion^8^. The size of KNM-BG 35250BE was suggested to approximate that of male *Papio cynocephalus*^8^. KNM-BG 35250BE shows the primitive condition for primates by retaining a bridge over the groove of the vertebral artery^8,29^. Subsequent discoveries of vertebral remains attributed to *Nacholapithecus kerioi* indicate that the superior articular facets of the atlas are rounded as in extant hominoids, and that the overall robusticity and large size of the cervical vertebrae could be related to the large forelimbs and head and the necessity to resist muscle reaction forces during locomotion^30^.

The aim of this contribution is to investigate the first cervical vertebra of *Otavipithecus namibiensis* and *Nacholapithecus kerioi* through comparative anatomy, linear measurements and geometric morphometric analyses, in order to detect functional signals. As such, in this study the atlases of *Otavipithecus namibiensis* and of *Nacholapithecus kerioi* are compared to those of 105 specimens of extant catarrhines (hominoids and cercopithecoids) and platyrrhines. Besides re-analyzing the dimensions and anatomy of the fossil atlases in a broader comparative context as compared to previous studies, here we present the first geometric morphometric analysis of Miocene ape atlases. Identified patterns are tentatively interpreted in a functional framework, adding to the body of evidence regarding the diversity of fossil hominoid postural and locomotor behaviours of Miocene apes.

## Results

### Comparative anatomy

**Fig. 1:**
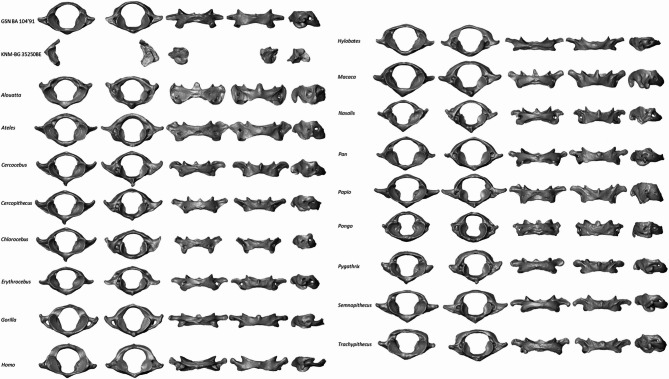
Comparative anatomy of primate atlases. Virtual renderings of the GSN BA 104’91 and KNM-BG 35250BE atlases compared to extant *Alouatta*, *Ateles*, *Cercocebus*, *Cercopithecus*, *Chlorocebus*, *Erythrocebus*, *Gorilla*, *Homo*, *Hylobates*, *Macaca*, *Nasalis*, *Pan*, *Papio*, *Pongo*, *Pygathrix*, *Semnopithecus* and *Trachypithecus* in superior, inferior, posterior, anterior and lateral left views. Images not to scale. Figure generated with Adobe Photoshop CS5.

The overall morphology of the atlas of GSN BA 104’91 and KNM-BG 35250BE was compared to those of *Alouatta*, *Ateles*, *Cercocebus*, *Cercopithecus*, *Chlorocebus*, *Erythrocebus*, *Gorilla*, *Homo*, *Hylobates*, *Macaca*, *Nasalis*, *Pan*, *Papio*, *Pongo*, *Pygathrix*, *Semnopithecus* and *Trachypithecus* (Fig. 1). Pictures from Mocke et al. (2022)^24^ were used to compare the morphology of GSN BA 13’21 with the sample investigated in the present study. The superior articular facets of GSN BA 104’91 and KNM-BG 35250BE are as concave as in the platyrrhines, most of the cercopithecoids, *Gorilla* and *Pan* but less than in *Papio* and *Pongo*. In GSN BA 104’91, the superior articular facets laterally cover the transverse foramina as in *Alouatta*, *Ateles*, *Erythrocebus*, *Homo*, *Hylobates*, *Nasalis*, *Pan*, *Papio*, *Pongo*, *Pygathrix*, *Semnopithecus* and *Trachypithecus*. The vertebral foramen in GSN BA 104’91 and GSN BA 13’2 is more extended mediolaterally than in *Gorilla* and *Homo* and resembles more closely the morphology of *Alouatta*, *Erythrocebus*, *Pan*, *Papio* and *Semnopithecus*. The transverse processes in GSN BA 104’91 are more anteriorly placed than in *Alouatta*, *Chlorocebus*, *Gorilla*,* Hylobates*, and *Nasalis*, and resembles the rest of the cercopithecoids (in particular *Papio*) and *Pan*. The transverse processes are more laterally extended than in *Alouatta*, *Chlorocebus*, *Homo* and *Pongo* but less than in *Ateles*, *Gorilla*, *Hylobates*, *Macaca*, *Papio* and *Semnopithecus*. In lateral view, the transverse processes are positioned more superiorly in GSN BA 104’91 than in *Alouatta*, *Ateles*, *Gorilla*, *Hylobates*, *Macaca*, *Pan*, *Papio* and *Pongo* and approximates the orientation observed in the rest of the cercopithecoids as well as *Homo* and *Pan*. In the three Miocene specimens, the inferior articular facets are relatively flat, as in the comparative hominids. In posterior view, the articular facets in GSN BA 104’91 and KNM-BG 35250BE are more vertically oriented than in *Ateles*, *Homo* and less than in *Papio*, and are similar to *Alouatta*, *Gorilla*, *Hylobates* and *Pan*. The retro-glenoid tubercles in GSN BA 104’91 project postero-medially, as in *Hylobates* and *Pan*, but are less prominent. The posterior arch in GSN BA 104’91 is narrower supero-inferiorly than in *Alouatta*, *Ateles*, *Homo*, *Hylobates*, *Pan*, *Pongo* and *Trachypithecus* but broader than in *Gorilla* and is more comparable to most of the cercopithecoids, in particular to *Papio*. The posterior arch of GSN BA 13’21 is incomplete. Unlike the comparative extant specimens, there is no posterior tubercle in both *Otavipithecus* specimens and the surface of the posterior arch is relatively smooth. In anterior view, the anterior arch in GSN BA 104’91 is broad supero-inferiorly and similar to the anterior arch of most of the cercopithecoids and *Pan* but narrower than in *Alouatta*, *Ateles*, *Hylobates* and *Pongo*. The anterior tubercle is less prominent than in the comparative specimens. GSN BA 104’91, and KNM-BG 35250BE lack prominent tubercles for attachment of the transverse occipital ligament^31^as in extant non-*Homo* hominoids. There are two *ponticulus posticus* in GSN BA 104’91 and one in GSN BA 13’21, as in the platyrrhines, cercopithecoids and *Pan* specimens included in Fig. 1 but no *ponticulus lateralis*.

### Linear measurements

**Fig. 2:**
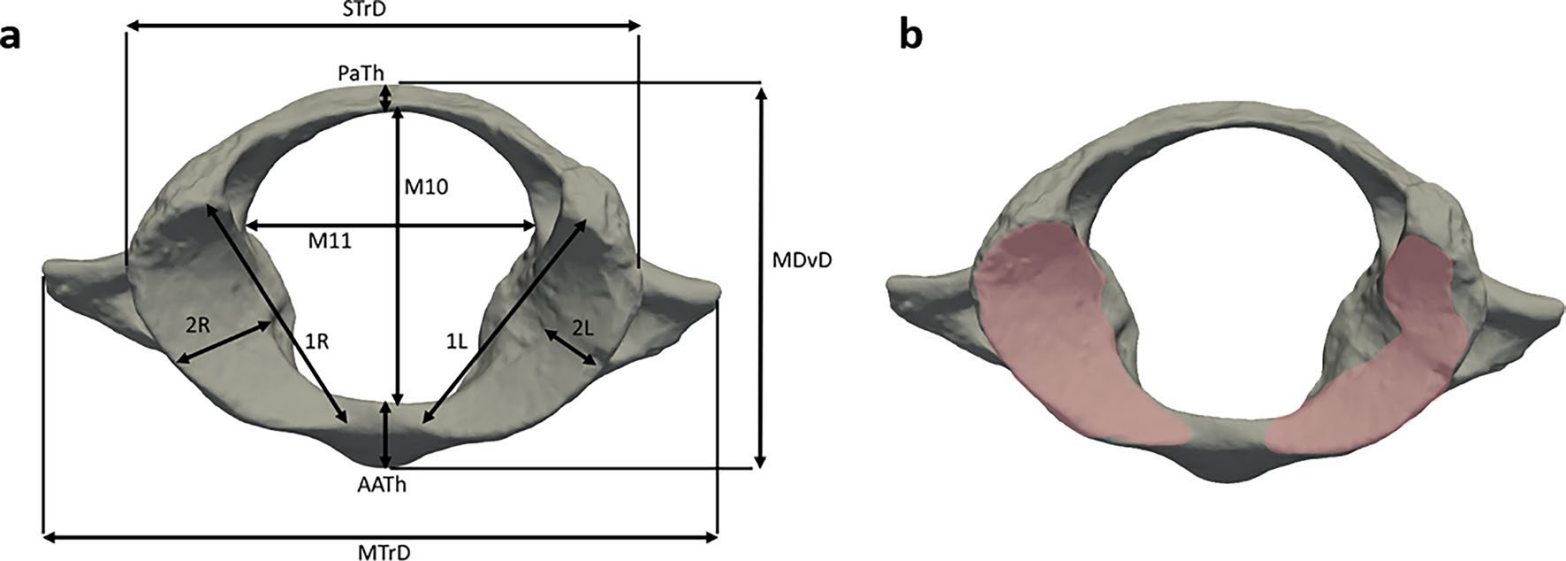
Methodological approach for capturing the dimensions of the atlas. Linear (**a**) and surface (**b**) measurements assessed in the atlas of GSN BA 104’91, KNM-BG 35250BE and of comparative specimens in superior view. AATh: anterior arch thickness; MDvD: maximum dorsoventral transverse diameter; MTrD: maximum transverse diameter; M10: canal dorsoventral maximum diameter; M11: canal transverse maximum diameter; PaTh: posterior arch thickness; STrD: superior transverse diameter; 1 L: diameter in major axis of the superior left articular facet; 2 L: diameter at a right angle to 1 L of the superior left articular facet; 1R: diameter in major axis of the superior right articular facet; 2R: diameter at a right angle to 1R of the superior right articular facet; 1 L/2L and 1R/2R: ratio between the diameter in the major axis and the orthogonal diameter. Figure generated with Adobe Photoshop CS5.

We used standard measurements published in Gómez-Olivencia et al. (2007)^17^ and Beaudet et al. (2020)^22^ for measuring the dimensions of the fossil and extant specimens (Fig. 2). To the exception of the superior articular facets, the overall dimensions of GSN BA 104’91 fall within the range of those of *Hylobates* and *Papio*, and are smaller than those of *Gorilla*, *Homo*, *Pan*, and *Pongo* but larger than those of *Alouatta*, *Ateles*, *Cercocebus*, *Cercopithecus*, *Chlorocebus*, *Erythrocebus*, *Macaca*, *Nasalis*, *Pygathrix*, *Semnopithecus* and *Trachypithecus* (Table 1). The size of the superior articular facets is overall similar to those of *Pan* and *Papio*, smaller than those of *Gorilla*, *Pongo* and *Homo* and larger than those of the rest of the sample. The diameter at a right angle to the diameter in major axis of the superior articular facet (2 L and 2R) is similar to the one measured in *Gorilla*, *Homo*, *Pan* and *Papio*. However, in terms of proportions (1R/2R and 1 L/2L), GSN BA 104’91 is overall smaller than the comparative specimens. Similarly, the measurements of GSN BA 13’21 mostly fall within the range of *Hylobates* and *Papio*. The dimensions of the right facet of KNM-BG 35250BE are similar to those of *Hylobates*, *Gorilla*, *Pongo*, *Pan* and *Papio*. In terms of surface area, the superior left articular facet of GSN BA 104’91 falls within the range of *Hylobates* and *Papio*, is smaller than *Pan*, *Gorilla*, *Pongo* and *Homo*, but is more extended than the other comparative groups and specimens. Similarly, the surface areas of the right articular facets of GSN BA 104’91 and KNM-BG 35250BE are in the range of *Hylobates* and *Papio*, smaller than *Pan*, *Gorilla*, *Pongo* and *Homo*, and higher than the rest of the comparative sample. Excluding the breadth of the superior articular facet of KNM-BG 35250BE, our measurements of the dimensions of GSN BA 104’91 and of KNM-BG 35250BE are overall consistent (i.e., less than 1 mm of differences) with those published in Conroy et al. (1996)^20^ and Nakatsukasa et al. (2007)^8^.

**Table 1:** Atlas osteological dimensions (in mm and mm^2^) of GSN BA 104’91 and KNM-BG 35250BE and comparative material. AATh: anterior arch thickness; ALF: area of the superior left articular facet; ARF: area of the superior right articular facet; mdvd: maximum dorsoventral transverse diameter; mtrd: maximum transverse diameter; M10: Canal dorsoventral maximum diameter; M11: Canal transverse maximum diameter; path: posterior arch thickness; s.d.: standard deviation; strd: superior transverse diameter; 1 L: diameter in major axis of the superior left articular facet; 2 L: diameter at a right angle to 1 L of the superior left articular facet; 1R: diameter in major axis of the superior right articular facet; 2R: diameter at a right angle to 1R of the superior right articular facet; 1 L/2L and 1R/2R: ratio between the diameter in the major axis and the orthogonal diameter.

Specimen/sample		AATh		MDvD		MTrD		M10		M11	PaTh		STrD		1 L		2 L		1 L/2L		ALF		1R		2R		1R/2R		ARF
GSN BA 104’91		3.9		26.4		46.2		20.8		20.0	2.1		32.5		16.6		10.3		1.6		90.7		17.0		9.4		1.8		108.2
GSN BA 13’21				23.0		42.0		16.0							15.8^‡^		3.7^‡^		4.3		-		15.8^‡^		3.7^‡^		4.3		-
KNM-BG 35250BE		-		-		-		-		-	-		-		-		-		-		-		13.6^¶^		7.3		1.9		104.6
Extant catarrhines																													
*Cercocebus galeritus* (*n* = 1)		3.6		20.5		39.7		14.8		15.1	2.3		25.0		12.0		6.2		1.9		75.7		13.5		6.0		2.3		75.3
*Cercocebus* sp. (*n* = 1)		3.3		16.4		30.5		12.3		12.8	1.3		20.2		9.0		3.8		2.4		35.2		9.3		4.2		2.2		40.3
*Cercopithecus diana* (*n* = 2)																													
mean		2.5		17.1		34.1		13.3		12.5	1.4		21.2		10.6		5.1		2.1		45.6		9.8		4.7		2.1		44.2
range		2.5–2.5		15.9–18.3		32.2–36.0		12.3–14.4		11.6–13.3	1.3–1.6		21.1–21.3		10.4–10.7		5.0-5.2		2.0-2.1		43.4–47.8		9.6–10.1		4.4-5.0		2.0-2.2		41.8–46.7
s.d.		0.0		1.7		2.6		1.5		1.2	0.2		0.1		0.2		0.1		0.1		3.2		0.4		0.5		0.1		3.4
*Cercopithecus neglectus* (*n* = 1)		3.1		17.1		36.9		12.7		12.9	1.4		21.9		10.5		5.0		2.1		48.0		10.0		5.6		1.8		46.2
*Chlorocebus aethiops* (*n* = 1)		2.1		14.8		29.1		11.0		11.6	2.1		19.5		9.5		5.5		1.7		38.3		9.7		4.2		2.3		43.9
*Erythrocebus patas* (*n* = 2)																													
mean		3.5		19.0		40.0		13.4		14.5	2.4		24.3		12.2		5.8		2.1		60.6		11.5		4.1		3.0		60.1
range		2.9–4.2		18.0-19.9		36.2–43.8		13.1–13.7		14.0-15.1	2.2–2.6		23.8–24.8		11.1–13.3		5.0-6.6		2.0-2.2		55.3–65.9		11.5–11.6		3.1-5.0		2.3–3.6		51.1–69.1
s.d.		0.9		1.3		5.4		0.4		0.8	0.3		0.7		1.5		1.2		0.2		7.5		0.1		1.3		0.9		12.8
*Gorilla gorilla* (*n* = 11)																													
mean		8.4		49.7		82.2		35.0		30.2	6.6		54.5		25.2		10.1		2.5		239.4		25.5		9.6		2.7		246.3
range		6.8–10.1		40.8–56.6		67.6-100.5		28.4–41.5		25.8–35.8	2.2–11.4		48.4–65.2		21.7–29.3		8.5–13.1		2.2-3.0		174.1-404.3		22.8–30.6		6.4–12.6		2.3–3.6		179.9-383.7
s.d.		1.1		5.5		11.3		3.9		3.1	3.8		5.7		2.6		1.6		0.3		68.9		2.7		1.8		0.4		71.3
*Gorilla beringei* (*n* = 7)																													
mean		6.6		49.1		84.1		35.4		27.4	6.9		54.8		22.8		10.6		2.2		218.9		21.8		11.5		1.9		219.6
range		5.7–8.6		43.7–54.6		74.5–99.2		33.1–40.0		23.5–33.9	3.1–10.6		50.9–58.2		18.2–26.9		9.0-12.6		1.7-3.0		162.9-278.1		19.0-23.9		8.4–13.6		1.6–2.7		167.9-270.3
s.d.		1.0		4.6		8.9		2.5		3.5	2.6		2.7		2.8		1.5		0.5		46.9		1.7		1.7		0.4		38.9
*Homo sapiens* (*n* = 10)																													
mean		5.8		42.2		72.4		29.8		27.4	6.9		48.2		22.8		10.7		2.1		187.0		22.3		9.8		2.3		185.9
range		4.4–8.1		38.2–44.5		64.6–78.4		27.4–32.2		26.0–30.0	3.4–8.5		40.6–52.7		19.4–26.3		8.5–13.4		1.7–2.6		149.1-216.7		20.1–24.9		8.3–11.7		1.9–2.9		152.3-216.7
s.d.		1.1		1.8		3.6		1.5		1.4	1.5		3.4		2.2		1.5		0.3		24.1		1.5		1.0		0.3		19.6
*Hoolock hoolock*		1.7		17.8		34.8		15.4		14.6	0.9		25.3		10.1		4.3		2.4		40.1		9.5		4.3		2.2		38.7
*Hylobates agilis* (*n* = 2)																													
mean		2.5		18.5		33.2		14.0		14.3	1.4		23.1		9.7		3.8		2.7		41.2		9.9		4.3		2.3		44.7
range		2.1–3.6		17.0–20.0		28.5–37.8		13.7–14.3		14.1–14.5	1.4–1.5		21.6–24.6		9.1–10.3		2.9–4.8		2.2–3.1		32.9–49.5		8.9–10.9		3.6–4.9		2.2–2.5		36.3–53.1
s.d.		0.8		2.1		6.6		0.5		0.3	0.0		2.1		0.9		1.3		0.7		11.7		1.4		0.9		0.2		11.9
*Hylobates klossi* (*n* = 1)		2.7		17.0		31.6		13.1		14.3	1.3		23.0		9.2		2.9		3.1		39.9		10.8		3.2		3.4		38.8
*Hylobates* lar (*n* = 2)																													
mean		3.1		20.4		36.7		14.7		14.2	2.6		26.2		11.0		4.8		2.3		66.9		11.4		5.0		2.3		73.8
range		2.2-4.0		17.9–22.9		33.0-40.3		14.6–14.8		13.5–14.8	1.0-4.1		24.6–27.8		10.9–11.0		4.2–5.3		2.1–2.6		40.3–93.6		11.3–11.5		4.3–5.6		2.0-2.7		49.0-98.6
s.d.		1.3		3.6		5.2		0.1		0.9	2.2		2.3		0.0		0.8		0.4		37.8		0.1		0.9		0.5		35.0
*Hylobates* sp. (*n* = 3)																													
mean		2.5		23.0		43.6		18.6		17.5	2.0		29.2		12.8		4.8		2.7		76.1		13.0		5.1		2.6		76.7
range		2.1–3.4		18.4–32.3		34.9–60.7		15.2–25.3		15.5–21.5	1.2–3.5		25.8–34.8		11.4–15.1		4.1–5.4		2.1–3.2		57.0-106.9		11.2–16.3		4.2–5.9		2.2–2.7		55.0-118.1
s.d.		0.8		8.1		14.8		5.9		3.4	1.3		4.9		2.0		0.6		0.5		27.0		2.9		0.9		0.3		35.9
*Macaca arctoides* (*n* = 1)		3.4		18.1		30.9		13.6		13.6	1.4		22.9		10.4		4.4		2.4		35.7		11.2		3.5		3.2		38.7
*Macaca fascicularis* (*n* = 1)		2.9		17.0		31.2		11.9		12.9	2.3		21.4		9.1		3.6		2.6		35.1		9.4		2.8		3.3		31.7
*Macaca fuscata* (*n* = 2)																													
mean		5.4		21.3		37.4		15.1		15.7	1.4		26.0		12.9		4.5		2.9		69.7		13.1		4.9		2.7		63.5
range		4.7–6.1		20.6–22.0		33.9–40.8		13.6–16.6		15.0-16.3	1.2–1.6		24.1–28.0		11.3–14.6		4.2–4.8		2.7-3.0		63.7–75.7		12.3–13.9		4.6–5.3		2.6–2.7		56.3–70.7
s.d.		0.9		1.0		4.9		2.2		0.9	0.3		2.7		2.3		0.4		0.2		8.5		1.1		0.5		0.0		10.2
*Macaca maura* (*n* = 1)		8.6		24.4		38.4		14.7		14.9	2.5		24.7		11.6		5.0		2.3		78.0		11.6		4.9		2.4		65.6
*Macaca mulatta* (*n* = 2)																													
mean		3.0		18.0		34.2		13.7		13.8	1.4		22.7		10.9		4.2		2.6		48.6		10.1		4.2		2.4		45.7
range		3.0–3.0		16.8–19.2		32.0-36.4		12.8–14.6		13.4–14.1	1.2–1.7		21.5–23.9		9.9–11.8		3.9–4.4		2.5–2.7		40.1–57.1		9.3–11.0		4.0-4.4		2.3–2.5		38.6–52.8
s.d.		0.0		1.7		3.1		1.3		0.5	0.3		1.7		1.3		0.3		0.1		12.0		1.3		0.3		0.1		10.0
*Nasalis larvatus* (*n* = 3)																													
mean		4.6		21.2		37.2		15.6		15.5	1.3		26.7		11.9		4.9		2.4		66.0		11.7		3.8		3.1		59.6
range		3.3–5.6		20.3–22.6		34.1–42.6		14.8–16.0		14.6–16.8	1.1–1.5		23.9–28.8		11.0-12.8		4.2–5.5		2.2–2.6		49.7–75.5		11.6–11.9		3.2–4.5		2.6–3.6		48.5–68.2
s.d.		1.2		1.2		4.7		0.7		1.2	0.2		2.5		0.9		0.7		0.2		14.2		0.2		0.7		0.5		10.1
*Pan paniscus* (*n* = 3)																													
mean		3.6		30.0		55.3		23.9		22.6	2.7		40.2		15.2		6.9		2.2		176.9		15.0		7.2		2.1		183.1
range		3.4–3.8		27.9–32.1		52.3–60.5		22.0-25.3		21.2–24.5	2.1–3.4		36.5–43.5		14.7–15.9		6.7–7.2		2.0-2.3		144.9-214.3		14.1–15.6		6.9–7.4		2.0-2.1		154.2-200.7
s.d.		0.2		2.1		4.6		1.7		1.7	0.6		3.5		0.6		0.3		0.1		35.0		0.7		0.3		0.0		25.3
*Pan troglodytes* (*n* = 11)																													
mean		5.3		35.5		64.8		25.0		23.9	4.9		45.7		19.0		8.0		2.4		221.3		19.8		7.8		2.6		241.7
range		3.2–6.9		31.9–39.7		59.9–69.5		23.0-27.6		20.8–27.8	3.4–7.4		39.0-49.6		15.8–21.2		6.6–10.1		1.8-3.0		146.5-279.1		17.3–26.6		6.4–11.5		1.6–3.7		163.1-340.6
s.d.		1.0		2.4		3.2		1.3		1.9	1.4		3.1		1.8		1.3		0.4		41.3		2.7		1.4		0.6		47.8
*Pan* sp. (*n* = 1)		3.5		29.8		45.2		23.0		21.6	3.0		37.9		16.6		6.4		2.6		221.1		15.4		7.1		2.2		223.4
*Papio anubis* (*n* = 5)																													
mean		4.4		26.2		54.8		19.5		19.8	2.8		32.9		17.8		7.4		2.6		155.1		17.7		7.3		2.6		155.2
range		3.4–5.4		21.6–37.5		43.5–74.3		16.2–28.0		16.9–27.4	1.6–4.6		29.3–43.1		13.5–24.7		3.8–12.7		1.9–3.5		80.9-296.8		15.1–23.8		4.0-12.2		2.0-3.8		95.5-286.3
s.d.		0.8		6.6		11.9		4.9		4.4	1.4		5.8		4.1		3.2		0.6		83.6		3.5		3.0		0.7		76.3
*Papio cynocephalus* (*n* = 1)		8.4		38.1		69.2		27.8		28.3	3.6		43.0		21.6		9.9		2.2		210.1		24.8		7.4		3.4		208.3
*Papio hamadryas* (*n* = 3)																													
mean		5.5		30.7		55.8		21.5		21.3	4.0		37.0		18.6		7.6		2.5		174.3		18.7		7.7		2.4		182.1
range		5.5–5.5		28.1–35.9		46.2–63.8		18.8–26.8		18.7–26.2	3.7–4.2		31.3–46.3		15.3–23.5		5.9–9.6		2.1–2.9		87.9-319.1		14.7–24.4		6.5–9.3		2.0-2.6		109.4-304.1
s.d.		0.0		4.5		8.9		4.6		4.2	0.3		8.1		4.3		1.9		0.4		126.2		5.0		1.5		0.3		106.3
*Pongo abelii* (*n* = 2)																													
mean		5.7		38.8		62.2		28.2		27.4	4.4		49.9		21.3		7.6		2.8		212.1		21.7		7.6		2.8		219.0
range		4.9–6.5		33.4–44.2		58.2–66.2		26.4–30.0		23.6–31.2	2.2–6.6		46.9–52.9		19.9–22.8		7.6–7.7		2.6-3.0		175.1-249.2		20.1–23.3		7.5–7.8		2.6–3.1		175.5-262.4
s.d.		1.1		7.6		5.7		2.6		5.4	3.2		4.3		2.0		0.1		0.2		52.4		2.2		0.2		0.4		61.5
*Pongo pygmaeus* (*n* = 8)		6.7		45.9		67.6		32.2		20.4	7.2		46.9		24.2		11.0		2.2		329.3		23.8		11.3		2.1		357.5
mean		5.2		37.1		60.1		28.7		23.1	3.7		47.9		21.1		9.4		2.3		238.3		21.7		9.4		2.3		247.5
range		3.5–6.4		32.4–41.1		48.3–74.0		26.2–31.0		19.9–27.6	1.4–4.6		40.5–60.2		18.4–23.8		7.4–11.4		2.0-2.6		152.3-339.1		18.0-24.8		6.7–11.5		2.0–3.0		145.0-357.5
s.d.		1.0		3.2		8.7		1.6		2.4	1.1		7.1		2.1		1.5		0.2		72.5		2.1		1.6		0.3		72.6
*Pygathrix nemaeus* (*n* = 1)		3.9		20.6		34.5		14.7		14.6	2.3		24.9		12.1		4.1		3.0		54.4		12.0		5.0		2.4		58.7
*Semnopithecus entellus* (*n* = 1)		4.0		19.3		38.0		14.0		13.6	1.7		24.4		10.2		3.3		3.1		42.8		10.5		3.6		2.9		51.7
*Symphalangus syndactylus* (*n* = 1)		3.0		22.5		38.8		17.1		16.3	2.3		28.5		13.0		4.5		2.9		54.4		12.8		3.9		3.3		53.2
*Trachypithecus francoisi* (*n* = 1)		3.7		18.5		34.5		12.7		14.4	2.0		23.4		11.5		5.3		2.2		52.7		10.9		6.6		1.7		54.5
*Trachypithecus obscura* (*n* = 1)		4.3		18.5		33.0		12.5		13.6	2.1		24.5		11.4		4.6		2.5		60.6		11.8		5.8		2.0		70.9
*Trachypithecus* sp. (*n* = 1)		2.0		14.6		24.6		12.0		11.0	1.1		18.2		8.8		3.4		2.6		27.9		8.7		4.1		2.1		29.0
Extant platyrrhines																													
*Alouatta palliata* (*n* = 2)																													
mean		4.0		17.4		33.8		12.0		13.3	1.9		22.9		11.1		3.1		3.7		41.7		9.6		3.0		3.2		34.2
range		3.3–4.7		15.8–19.0		27.0-40.5		12.0–12.0		12.1–14.4	1.0-2.9		19.6–26.2		10.4–11.9		2.3–3.9		3.0-4.5		31.5–51.9		9.5–9.6		2.9–3.1		3.1–3.3		31.7–36.7
s.d.		1.0		2.3		9.5		0.1		1.6	1.3		4.6		1.1		1.2		1.0		14.5		0.1		0.2		0.2		3.5
*Alouatta seniculus* (*n* = 3)																													
mean		4.0		18.0		31.5		12.4		13.2	1.8		24.8		10.4		4.2		2.5		58.7		10.7		3.6		3.0		52.4
range		3.6–4.3		17.1–18.6		27.8–37.7		11.4–13.1		12.3–14.4	1.4–2.5		24.1–26.0		9.7–11.2		3.5–4.9		2.1–3.2		54.2–65.4		9.2–11.8		3.4–3.8		2.5–3.5		47.0-62.6
s.d.		0.3		0.8		5.4		0.9		1.1	0.6		1.1		0.8		0.7		0.6		6.0		1.3		0.2		0.5		8.8
*Ateles fusciceps* (*n* = 4)																													
mean		2.5		18.8		37.1		14.6		14.5	2.1		26.7		11.0		4.6		2.5		53.7		11.4		4.4		2.7		55.9
range		2.2–2.9		17.2–19.8		35.1–38.9		12.4–15.9		12.9–15.9	1.6–2.6		24.8–28.4		10.1–12.6		3.9–5.3		1.9–3.2		41.7–61.8		9.9–12.6		3.7-5.0		2.1–3.4		49.0-62.6
s.d.		0.3		1.2		1.9		1.6		1.3	0.4		1.6		1.1		0.6		0.6		9.5		1.1		0.6		0.6		5.7

^‡^Here we use the means for the left and right articular facets provided by Mocke et al. (2022).

^§^Estimate based on the reconstruction of the left transverse process (see Beaudet et al., 2020).

^¶^Since the articular facet is not complete, here we provide a minimum estimate only.

### Geometric morphometric analyses

**Fig. 3:**
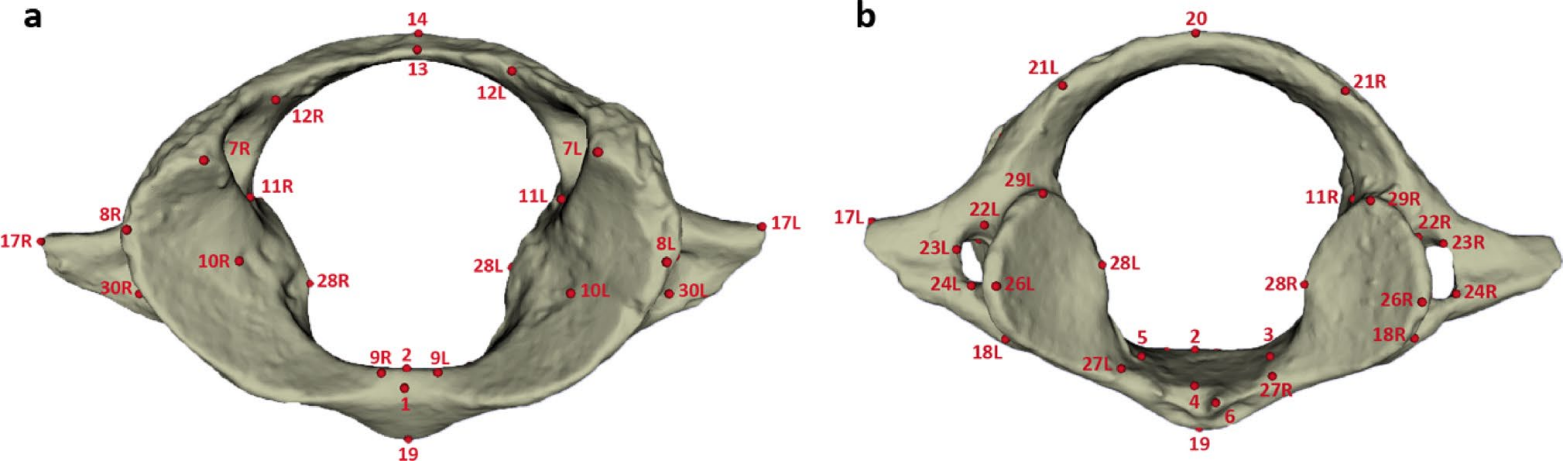
Landmark set. Set of landmarks positioned along the external surface of the atlas of GSN BA 104’91 in superior (**a**) and inferior (**b**) views. Figure generated with Adobe Photoshop CS5.

**Fig. 4:**
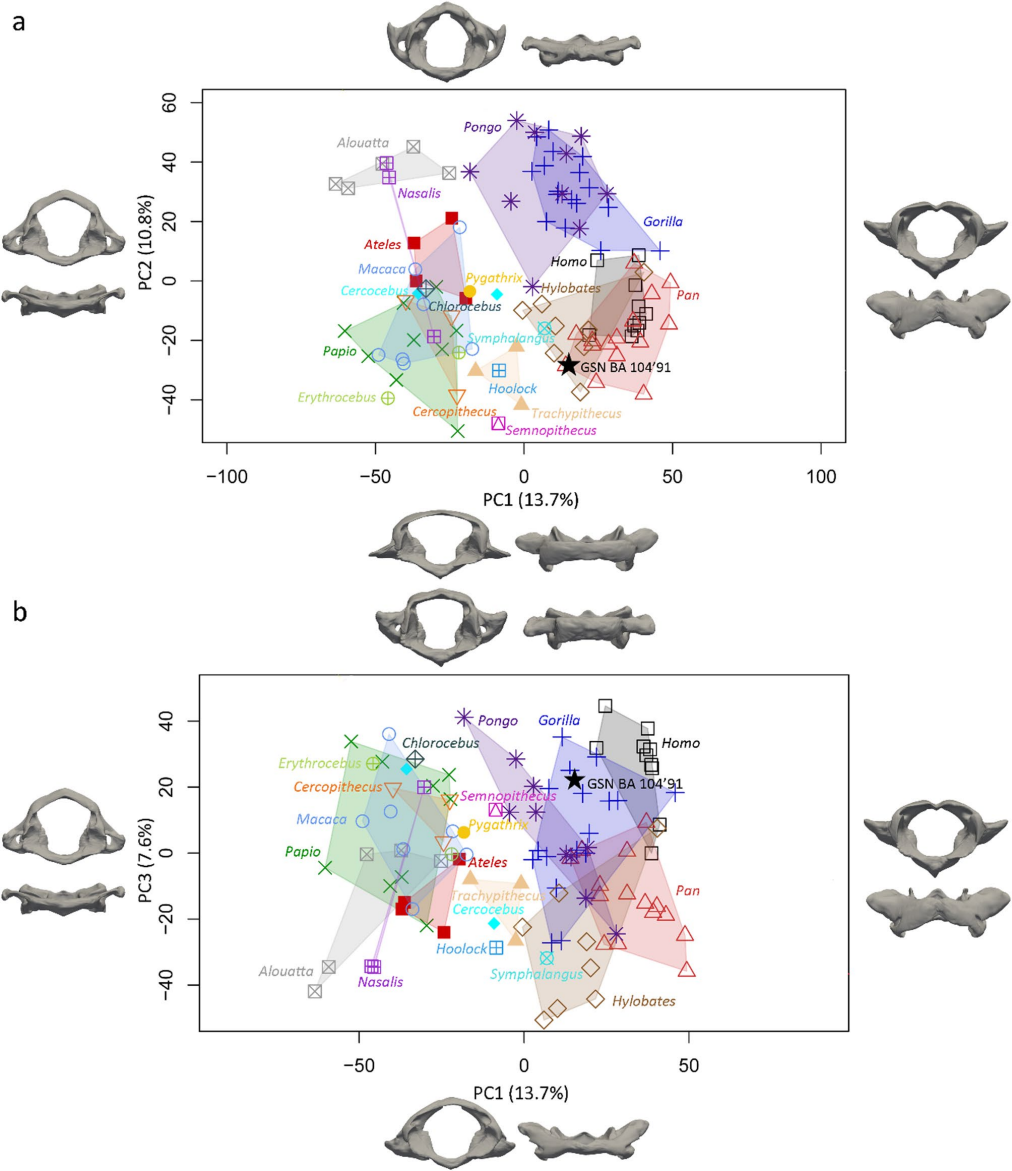
Quantitative analysis of shape variation in complete atlases. Principal component analysis (PCA) of the Procrustes-registered shape coordinates of the complete atlas morphology calculated for GSN BA 104’91 and comparative extant primates for PC1, PC2 (**a**) and PC3 (**b**). Shapes at the extremes of the axes illustrate morphological variation trends along each component in superior and posterior views. Figure generated with RStudio 1.4.1106.

The morphology of the fossil and extant specimens was comparatively and quantitatively assessed through a landmark-based three-dimensional geometric morphometric approach following the protocol detailed in Manfreda et al. (2006)^13^ (Fig. 3). Figure 4 shows the PCA performed on the Procrustes shape coordinates of the complete atlases and the distribution along PC1 and PC2 (Fig. 4A) and PC1 and PC3 (Fig. 4B). Two clusters of extant primates are identified along PC1 (13.7% variance explained) that roughly discriminate hominoids from the other primates. The hominoid specimens plot in the positive space of PC1 (Fig. 4A), that corresponds to a rounded vertebral canal, antero-posteriorly extended articular facets, and laterally and supero-inferiorly extended transverse processes that are positioned posteriorly, while the rest of the specimens are found in the negative space, that corresponds to a medio-laterally compressed vertebral canal, rounded transverse foramina and small transverse processes. Along PC2 (10.8% variance explained, Fig. 4A), the *Cercocebus*, *Cercopithecus*, *Chlorocebus*, *Erythrocebus*, *Homo*, *Hoolock*, *Hylobates*, *Pan*,* Papio*,* Pygathrix*,* Semnopithecus*, *Symphalangus*, *Trachypithecus* specimens fall in the negative space, that corresponds to transverse processes positioned more anteriorly and articular facets that are medio-laterally compressed, while the *Alouatta*, *Ateles*, *Gorilla*, *Nasalis*, *Hylobates*, and *Pongo*, specimens fall in the positive space, that corresponds to transverse processes positioned more posteriorly and antero-posteriorly elongated articular facets and transverse processes. Figure 4B shows the distribution along PC1 and PC3. Along PC3 (7.6% variance explained), the *Alouatta*,* Cercocebus*, most of *Hylobates* and *Pan and Nasalis*, Hoolock, part of *Papio*, *Symphalangus* and *Trachypithecus* specimens fall in the negative space, that corresponds to a medio-laterally wide vertebral canal, and short transverse processes that are positioned antero-inferiorely, while the *Cercopithecus*, *Chlorocebus*, *Erythrocebus*, most of *Gorilla* and *Pongo* and *Papio* and *Macaca*, *Homo*, *Pygathrix*, *Semnopithecus*, specimens cluster in the positive space, that corresponds to an antero-posteriorly elongated vertebral canal, and short and medio-laterally compressed transverse processes. In both plots, GSN BA 104’91 falls within the cluster of extant hominoids, and particularly close to *Gorilla*,* Hylobates* and *Pan*. Procrustes distances computed between *Otavipithecus* and the mean shapes of extant groups indicate closest morphological similarity to *Pan* (Table S3). The PCA that focuses on hominoids further confirms similarities with *Pan* (Figure S1).

**Fig. 5:**
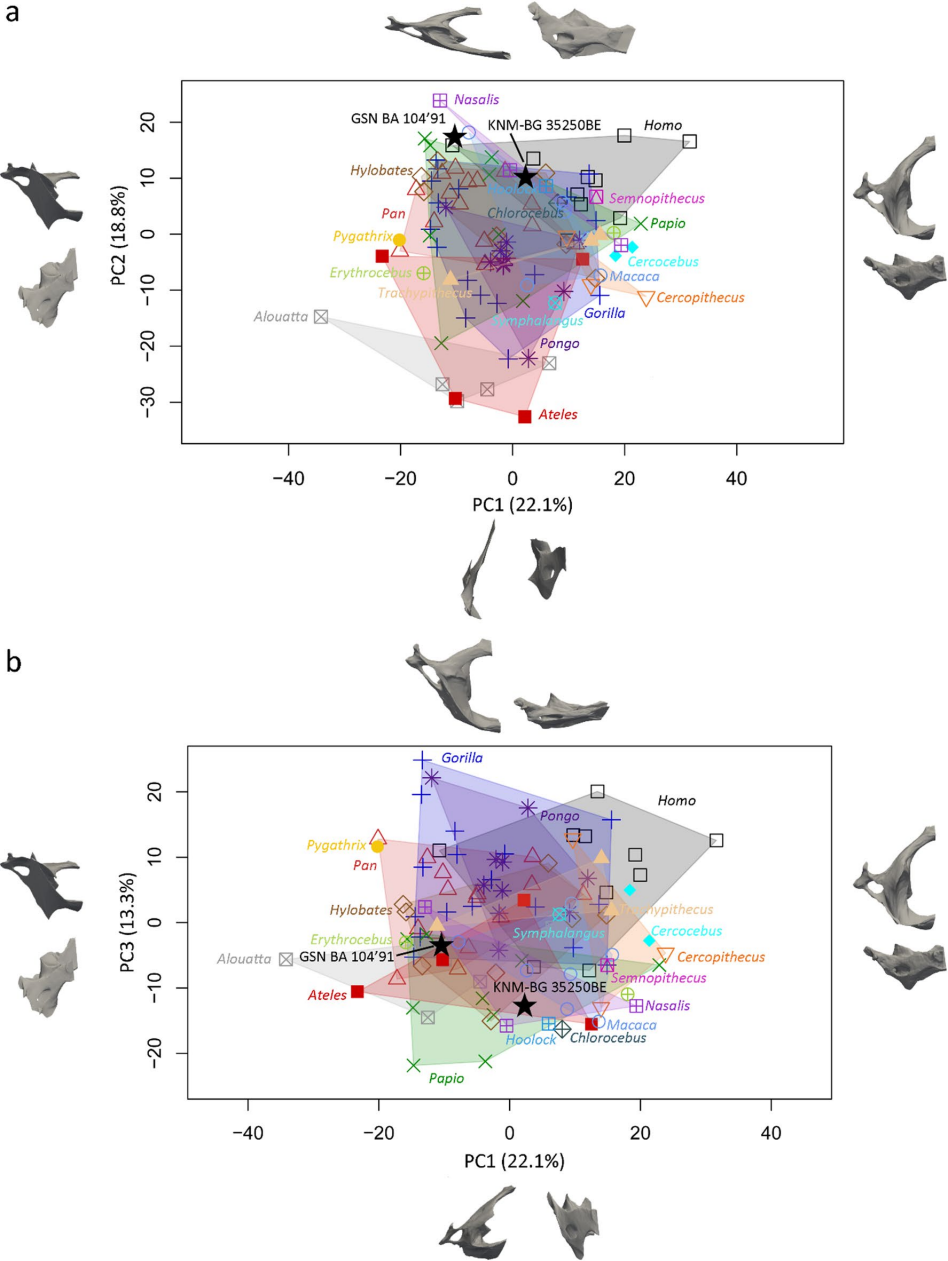
Quantitative analysis of shape variation in partial atlases. Principal component analysis (PCA) of the Procrustes-registered shape coordinates of the partial atlas morphology calculated for GSN BA 104’91 and KNM-BG 35250BE, and comparative extant primates for PC1, PC2 (**a**) and PC3 (**b**) using 9 selected landmarks. Shapes at the extremes of the axes illustrate morphological variation trends along each component in superior and posterior views. Figure generated with RStudio 1.4.1106.

Figure 5 shows the PCA performed on the Procrustes shape coordinates of the incomplete atlases (i.e., landmarks retained correspond to the preserved region of KNM-BG 35250BE). Along the two first axes (Fig. 5A), the extant groups mostly overlap. There is no clear trend along PC1 (representing 22.1% variance explained), as most of the clusters are distributed across both negative and positive values. Along PC2 (18.8% variance explained), the *Alouatta*, *Ateles*, *Cercocebus*, *Cercopithecus*, *Erythrocebus*, *Symphalangus*, and *Trachypihtecus* specimens plot in the negative space, that represents a medio-laterally compressed articular facet and a short transverse process. The rest of the sample, mostly hominoids, fall within the positive space and are characterized by an antero-posteriorly compressed atlas, an articular facet that is extended antero-posteriorly, a transverse process positioned at the same level as the articular facet with a medio-laterally extended oval transverse foramina. Along PC3 (13.3% variance explained, Fig. 5B), most of the hominoid specimens, as well as *Pygathrix*, and *Trachypithecus*, cluster in the positive space, that corresponds to an articular facet elongated antero-posteriorly and a transverse process positioned posteriorly with an oval foramen. The rest of the specimens plot in the negative space, that corresponds to an antero-posteriorly compressed atlas positioned at the same level as the articular facet with a rounded foramen. GSN BA 104’91 and KNM-BG 35250BE falls within or close to the clusters of hominoids and *Papio*, which is further confirmed by the Procrustes distances (Table S4). Procrustes distances suggest additional morphological affinities with *Nasalis*. The PCA that only includes hominoids as comparative sample reveal similarities of GSN BA 104’91 and KNM-BG 35250BE with *Pan*, *Gorilla* and *Hylobates* when the three components are considered (Figure S2).

## Discussion

The overall size of GSN BA 104’91 is similar to *Hylobates* and *Papio* and smaller than the one of *Gorilla*, *Homo*, *Pan*, and *Pongo* but larger than the one of the platyrrhines and the rest of the catarrhines investigated in this study. However, the size, proportion and surface area of the articular facets of GSN BA 104’91 and KNM-BG 35250BE approximate the measurements reported for *Hylobates*, *Pan* and *Papio*. Discrepancies between the measurements reported in the present study and previous conclusions inferred from the comparison of the dimensions of the atlas of GSN BA 104’91 and KNM-BG 35250BE and those of extant primates^8,20,32^ could be tentatively explained by differences in the composition of the comparative sample (e.g., more individuals in the present study, additional taxa represented). When the overall morphology of the atlas is considered, GSN BA 104’91 is similar to the extant hominoids, and in particular to *Pan* when the hominoids only are considered (Fig. 4, Figure S1). The morphology of the right lateral mass of GSN BA 104’91 and KNM-BG 35250BE approximates the condition of the extant hominoids and *Papio* (Fig. 4, Figure S1).

Within the limit of our sample, our morphometric analysis of complete atlases tentatively separates hominoid from non-hominoid taxa (Fig. 4). In particular, the hominoid morphotype identified in this study is characterized by a medio-laterally compressed vertebral canal and small transverse processes, while the non-hominoid morphotype is characterized by a rounded vertebral canal, antero-posteriorly extended articular facets, and laterally and supero-inferiorly extended transverse processes that are positioned posteriorly. If the patterns identified do reflect positional behaviors (but see below), similarities with extant hominoids, and more particularly with *Pan*, revealed by our analysis might be informative for reconstructing the positional behaviors of *Otavipithecus* if considered in conjunction with functional signals that derive from the study of the rest of the skeleton. While it is not possible to identify the nature of the behaviors represented in the repertoire of *Otavipithecus* and shared with *Pan* (e.g., terrestrial quadrupedalism, climbing), possible morphological affinities detected in the lateral mass of *Otavipithecus* with *Papio* could support the presence of terrestrial activities in the positional behavior of this fossil genus^23,24^.

Intriguingly, areas of insertion for the attachments of muscles and ligaments are not particularly enlarged in GSN BA 104’91 (Fig. 1), which could indicate reduced muscle mass (e.g., rectus capitis posterior minor that originates from the posterior tubercle, and the superior oblique portion of the longus colli that originates from the anterior tubercles) and/or a vestigial form of the ligament essential for head stabilization during locomotion (i.e., nuchal ligament attached to the posterior tubercle)^11^. As such, it is likely that the lack of strong muscle and ligament attachments reflects a weaker demand on the muscular system to ensure head stability in *Otavipithecus*, on the contrary to obligate bipeds^33^even though the atlas of *Homo* might reflect adaptations related to other aspects of their postural and locomotor repertoire. Moreover, the close similarities of the orientation of the transverse processes between *Otavipithecus* and *Pan* is also informative for reconstructing the musculoskeletal system of this extinct taxon, especially since specificities exist in extant apes in general, and *Pan* in particular. For example, the muscle that connects the transverse processes of the third and first cervical vertebrae is only found in *Pan*, and the atlanto-clavicularis muscle that runs from the transverse processes of the atlas to the clavicle is present in apes but absent in humans^16^. While we cannot say with certainty that these muscles were also present in *Otavipithecus*, this is a possibility to consider.

Discriminating primate taxa or positional behaviors is not possible when restricting the morphometric study to the right lateral mass (Fig. 5). However, through this approach similarities between *Otavipithecus* and extant *Papio* were detected and confirm the presence of cercopithecoid-like features as reported by the first description of GSN BA 104’91 by Conroy et al. (1996)^20^. Contrasted signals detected in the study of the entire atlas and of the right lateral mass only might reflect the differential nature of the demands applied to the articular facets (i.e., biomechanical) and the vertebral foramen (i.e., neurovascular). The morphological affinities of KNM-BG 35250BE as quantified by our comparative analysis of the right lateral mass is less clear. Indeed, our study indicates the presence of a mix of hominoid-like and cercopithecoid-like, which is consistent with the conclusion of Kikuchi et al. (2012)^30^ that reported intermediate features between extant great apes and other primates. Moreover, previous studies of the *Nacholapithecus* vertebral morphology raised the possibility of unique functional adaptation and arboreal behaviors in this taxon^8,9,30,32,34–39^. However, as illustrated with the more complete atlas of *Otavipithecus*, morphological affinities as quantified by GM and PCA analyses vary depending on if the overall atlas or only the lateral mass is investigated (Figs. 4 and 5). In this respect, the morphometric analysis of more complete atlases of *Nacholapithecus* would be crucial. Although previous studies have identified correlations between morphological features of the atlas and primate locomotor patterns^13^, we must acknowledge the fact that patterns detected in our analysis may partly reflect phylogenetic relationships. In particular, hominoids are distinguished from platyrrhines and the other catarhines in our sample. Phylogeny thus represents a potential confounding factor when interpreting musculoskeletal features within a functional perspective, especially given that primate soft-tissue anatomy has been shown to align closely with molecular phylogenies, thereby reflecting phylogenetic relationships^40^. Accordingly, our functional interpretations should be viewed as tentative and interpreted with caution. That said, if our results indeed reflect phylogenetic signal, they support the interpretation of *Otavipithecus* as the oldest known hominoid representative in southern Africa and highlight the taxonomic relevance of cervical vertebrae. Moreover, while previous studies have identified allometric patterns in the primate atlas, specifically correlations between body size and features such as the orientation of the transverse processes and the configuration of posterior arches^13,14^, morphological differences highlighted in our study do not center on those structures (e.g., proportions of the articular facets, shape of the vertebral canal). As such, allometric effects are unlikely to account for the overall variation we observed.

## Materials and methods

### Materials

GSN BA 104’91, found in a bone-bearing breccia block from Berg Aukas (Namibia), is a virtually complete atlas attributed to *Otavipithecus namibiensis* and curated at the Geological Survey of Namibia (Fig. 1)^20^. Fossil remains from Berg Aukas are dated biochronologically to 12–13 Ma^20,25^. KNM-BG 35250BE only preserves the right lateral mass and base of the posterior arch of the atlas and is part of the *Nacholapithecus kerioi* skeleton discovered at the site BG-K of Nachola (Kenya), and dated to 15 Ma (Fig. 1)^32,41,42^. KNM-BG 35250BE is housed at the National Museums of Kenya.

For comparative material, we included the measurements and descriptions published in Mocke et al. (2022)^24^ of the newly discovered atlas GSN BA 13’21 from Berg Aukas attributed to *Otavipithecus* but we were not able to digitize nor to perform new analyses on this specimen. Our comparative sample of extant catarrhines and platyrrhines comprised 105 atlases of non-pathological adult *Alouatta seniculus* (*n* = 3), *A. palliata* (*n* = 2), *Ateles fusciceps* (*n* = 4), *Cercocebus galeritus* (*n* = 1), *Cercocebus* sp. (*n* = 1), *Cercopithecus diana* (*n* = 2), *C. neglectus* (*n* = 1), *Chlorocebus aethiops* (*n* = 1), *Erythrocebus patas* (*n* = 2), *Gorilla gorilla* (*n* = 11), *G. beringei* (*n* = 7), *Homo sapiens* (*n* = 10), *Hoolock hoolock* (*n* = 1), *Hylobates agilis* (*n* = 2), *H. klossii* (*n* = 1), *H. lar* (*n* = 2), *Hylobates* sp. (*n* = 3), *Macaca arctoides* (*n* = 1), *M. fascicularis* (*n* = 1), *M. fuscata* (*n* = 2), *M. maura* (*n* = 1), *M. mulatta* (*n* = 2), *Nasalis larvatus* (*n* = 3), *Pan troglodytes* (*n* = 11), *P. paniscus* (*n* = 3), *P.* sp. (*n* = 1), *Papio anubis* (*n* = 7), *Pa. cynocephalus* (*n* = 1), *Pa. hamadryas* (*n* = 1), *Pongo abelii* (*n* = 2), *Po. Pygmaeus* (*n* = 8), *Pygathrix nemaeus* (*n* = 1), *Semnopithecus entellus* (*n* = 1), *Symphalangus syndactylus* (*n* = 1), and *Trachypithecus francoisi* (*n* = 1), *T. obscura* (*n* = 1), *T. sp.* (*n* = 1) sampling males and females (Table S1). Part of our comparative specimens were accessed on MorphoSource (www.morphosource.org, *n* = 54). Positional behaviours of extant taxa are detailed in Table S1^43–46^.

### Virtual reconstructions

3D models of GSN BA 104’91 and KNM-BG 35250BE were generated using photogrammetry and an Artec Space Spider 3D scanner, respectively. Most of the comparative extant specimens have been downloaded from MorphoSource (https://www.morphosource.org/) and from the Digital Morphology Museum KUPRI (http://dmm.pri.kyoto-u.ac.jp/dmm/WebGallery/dicom/researcherTop.html; Supplementary Table S1) and were imaged by X-ray tomography using various systems (Supplementary Table S1). Four of the extant human specimens have been rendered by using Next Engine laser scanner (Pretoria, South Africa).

### Linear measurements

We measured the dimensions of GSN BA 104’91, KNM-BG 35250BE and the extant comparative specimens following the protocol published in Gómez-Olivencia et al. (2007)^17^ (Fig. 2). Abbreviations of the measurements are detailed in Gómez-Olivencia et al. (2007)^17^ and Fig. 2 of the present study. Measurements were performed either physically with a digital caliper or virtually using Avizo v9.0 (Visualization Sciences Group Inc.). Not all of the measurement could be taken from the incomplete specimens KNM-BG 35250BE. For GSN BA 13’21, we used the measurements published in Mocke et al. (2022)^24^ to the exception of the anterior arch thickness (AATh), canal transverse maximum diameter (M11), posterior arch thickness (PaTh) and superior transverse diameter (STrD)^17^ that were not published. A ratio of the length and breadth of each facet was acquired by dividing the diameter in the major axis by the orthogonal diameter as a mean to describe the proportions and configuration of the articular facets (i.e., more extended medio-laterally or postero-anteriorly^22^). Moreover, the areas of the superior articular facets, that represent the areas in contact with the occipital condyles, were measured by manually isolating the articular facets in Avizo v9.0.

### Geometric morphometrics analyses

The overall morphology of the Miocene and extant comparative atlases was quantitatively investigated using landmark-based three-dimensional geometric morphometric approach following the protocol detailed in Manfreda et al. (2006)^13^. A total of 56 type II and type III landmarks were positioned on the external surface of all atlases (Fig. 3) using the software 3D Slicer 5.2.2. (http://www.slicer.org) and the tool “Control Point”^47^; https://www.slicer.org/). Because KNM-BG 35250BE is incomplete, we ran a second analysis based on 9 landmarks (7R, 8R, 10R, 11R, 18R, 26R, 27R, 28R, 29R) that correspond to the regions that are preserved in this specimen (Fig. 1). We computed a generalized Procrustes analysis^48^ using RStudio 1.4.1106 (RStudio Team, 2019) and the package ‘Morpho’^49^ (version 2.9). We performed a principal component analysis (PCA) to investigate shape variation within our comparative sample. The fossil specimens were then projected onto the analysis. In addition to computing PCAs with the entire comparative sample, we generated another PCA by excluding non-hominoid specimens from our comparative sample to investigate more specifically similarities/differences between extant and fossil hominoid specimens (see Supplementary Information). Shape variation was visualized by using the ‘warpRefMesh’ and ‘plotRefToTarget’ functions from the ‘geomorph’ package (version 4.0.0) to warp the mean shape into the maximum and minimum mean values for each axis^50,51^. Lastly, we computed Procrustes distances between the fossil specimens and the extant group means.

## Electronic supplementary material

Below is the link to the electronic supplementary material.

Supplementary Material

## Data Availability

The data that support the findings of this study (i.e., 3D models) are available upon request from E. Gilissen (Royal Museum for Central Africa), Y. Kikuchi (Saga University), G. Krüger (University of Pretoria), H. Mocke (Geological Survey of Namibia), E. Ndiema (National Museums of Kenya), and Bernhard Zipfel (University of the Witwatersrand), and but restrictions apply to the availability of these data, which were used under license for the current study, and so are not publicly available. Data are however available from the authors upon reasonable request and with permission of E. Gilissen (Royal Museum for Central Africa), Y. Kikuchi (Saga University), G. Krüger (University of Pretoria), H. Mocke (Geological Survey of Namibia), E. Ndiema (National Museums of Kenya), and Bernhard Zipfel (University of the Witwatersrand). Part of our comparative specimens were accessed on MorphoSource (www.morphosource.org).
