# Supplementary material for: New insights into the first cervical vertebrae of Otavipithecus and Nacholapithecus
Source: Sci Rep. 2025 Jul 8;15:24569. doi: 10.1038/s41598-025-09006-x (PMC12238335; doi:10.1038/s41598-025-09006-x)
Supplement: Supplementary file 1 — Supplementary Material [file 41598_2025_9006_MOESM1_ESM.docx]

**Supplementary information**

**Table S1.** Comparative sample used for assessing variation in osteological dimensions and morphology of the atlas. AMNH: American Museum of Natural History, New York, USA; CASHP: Center for Advanced Study of Human Paleobiology, George Washington University, USA; CM: Cleveland Museum, USA; DID: Diagnostic Imaging Department of the NC State Veterinary Hospital, USA; DU: Department of Evolutionary Anthropology, Duke University, USA; EHUB: Center for the Evolutionary Origins of Human Behavior, Kyoto University, Japan; ESI: Evolutionary Studies Institute, South Africa; JMC: Japan Monkey Centre, Inuyama, Japan; MAL: Museo di Archeologia Ligure, Genova, Italy; Necsa: South African Nuclear Energy Corporation, Pelindaba, South Africa; PBC: Pretoria Bone Collection, University of Pretoria, South Africa; RMCA: Royal Museum for Central Africa, Tervuren, Belgium; SMiF: Shared Materials Instrumentation Facility, Duke University, USA; SBUMC: Stony Brook University Medical Center, Stony Brook Universit, New York, USA; UA: Department of Anthropology, University of Arkansas, USA; UGCT: Centre for X-ray Tomography of Ghent University, Belgium; YPBM: Peabody Museum of Natural History, Yale University, USA; Y-TRIC: Yale Transitional Research Imaging Center, USA. F: female; M: male; ?: no information available; *: contain specimens downloaded from MorphoSource. Locomotor and postural behaviors are listed from the most to the less frequent and are based on Hunt (1991), Gebo (1996), Hunt et al. (1996) and Estrada and Marshall (2024).

| Specimen/Sample |  | Taxonomic  attribution | Sex | Stored at | Imaging facility | Locomotion | Posture |
| --- | --- | --- | --- | --- | --- | --- | --- |
| Extant catarrhines | |  |  |  |  |  |  |
| *Cercocebus* (*n* = 2)* |  | *Cercocebus galeritus* | M (*n* = 1) | EHUB | EHUB | Arboreal quadrupedalism, terrestrial quadrupedalism, leaping, clambering | Pronograde standing, sitting, crouching |
|  |  | *Cercocebus* sp. | M (*n* = 1) | UA | UA |  |  |
| *Cercopithecus* (*n* = 3) |  | *Cercopithecus diana*  *Cercopithecus neglectus* | F (*n* = 1)  M (*n* = 1)  M (*n* = 2) | EHUB | EHUB | Arboreal quadrupedalism, leaping, clambering | Pronograde standing, sitting, crouching |
| *Chlorocebus* (*n* = 1) |  | *Chlorocebus aethiops* | M (*n* = 1) | EHUB | EHUB | Quadrupedal walking and running, climbing, leaping | Sitting, standing, suspensory |
| *Erythrocebus* (*n* = 2) |  | *Erythrocebus patas* | F (*n* = 1)  M (*n* = 1) | EHUB | EHUB | Quadrupedal running, quadrupedal walking, climbing | Sitting, standing |
| *Gorilla* (*n* = 18)* |  | *Gorilla beringei* (*n* = 7)  *Gorilla gorilla* (*n* = 11) | F (*n* = 2)  M (*n* = 6)  F (*n* = 4)  ? (*n* = 1) | RMCA  EHUB AMNH  YPM  AMNH  AMNH  AMNH | UGCT  EHUB CASHP  Y-TRIC  SBUMC  CASHP  AMNH | Terrestrial quadrupedalism (knuckle-walking), climbing, bipedalism | Sitting, suspensory postures, standing |
| *Homo* (*n* = 10)* |  | *Homo sapiens* | F (*n* = 5)  M (*n* = 3)  ? (*n* = 2) | PBC  DU  PBC  MAL | Necsa  SMiF  PBC  MAL | Habitual terrestrial bipedalism | Orthograde standing, sitting and lying |
| *Hoolock* (n = 1)* |  | *Hoolock hoolock* | F (*n* = 1) | AMNH | AMNH | Brachiation, vertical climbing, leaping, bipedal walking | Sitting, suspensory postures |
| *Hylobates* (n = 8)* |  | *Hylobates agilis* (*n* = 2)  *Hylobates klossii* (*n* = 1)  *Hylobates lar* (*n* = 2)  *Hylobates* sp. (*n* = 3) | M (*n* = 3)  F (*n* = 2)  ? (*n* = 3) | EHUB DU  AMNH  AMNH  AMNH AMNH | EHUB SMiF  DID  CASHP  SBUMC AMNH | Brachiation, vertical climbing, leaping, bipedal walking | Sitting, suspensory postures |
| *Macaca* (*n* = 7)* |  | *Macaca arctoides* (*n* = 1)  *Macaca fascicularis* (*n* = 1)  *Macaca fuscata* (*n* = 2)  *Macaca maura* (*n* = 1)  *Macaca mulatta* (*n* = 2) | F (*n* = 3)  M (*n* = 4) | EHUB AMNH AMNH | EHUB CASHP  AMNH | Quadrupedal walking, climbing, leaping | Sitting, standing, suspensory |
| *Nasalis* (*n* = 3)* |  | *Nasalis larvatus* (*n* = 3) | F (*n* = 2) M (*n* =) | JMC AMNH | EHUB CASHP | Quadrupedal walking and clambering, leaping, climbing | Sitting, suspensory postures |
| *Pan* (*n* = 15)* |  | *Pan* *paniscus* (*n* = 3)  *Pan* sp. (*n* = 1)  *Pan* *troglodytes* (*n* = 11) | F (*n* = 8)  M (*n* = 5)  ? (*n* = 2) | RMCA  EHUB AMNH | UGCT  EHUB DID | Terrestrial quadrupedalism (knuckle-walking), climbing, bipedal walking | Sitting, suspensory postures, standing |
| *Papio* (*n* = 9)* |  | *Papio* *anubis* (*n* = 7)  *Papio* *cynocephalus* (*n* = 1)  *Papio* *hamadryas* (*n* = 1) | F (*n* = 3)  M (*n* = 3)  ? (*n* = 3) | RMCA  JMC AMNH AMNH | UGCT  EHUB CASHP  AMNH | Terrestrial quadrupedalism, arboreal quadrupedalism, leaping | Sitting, pronograde standing, lying |
| *Pongo* (*n* = 10)* |  | *Pongo abelii* (*n* = 2)  *Pongo pygmaeus* (*n* = 8) | F (*n* = 6) M (*n* = 4) | EHUB AMNH  UA | EHUB CASHP  UA | Orthograde suspensory, quadrupedalism, climbing, bipedalism | Orthograde suspensory, standing, sitting |
| *Pygathrix* (*n* = 1) |  | *Pygathrix nemaeus* (*n* = 1) | M (*n* = 1) | EHUB | EHUB | Arm-swinging (brachiation), quadrupedal walking/running | Forelimb suspensory postures, sitting |
| *Semnopithecus* (*n* = 1) |  | *Semnopithecus entellus* (*n* = 1) | F (*n* = 1) | JMC | EHUB | Terrestrial quadrupedal walking, climbing, leaping | Sitting, standing, suspensory |
| *Symphalangus* (*n* = 1) |  | *Symphalangus syndactylus* (*n* = 1) | M (*n* = 1) | AMNH | AMNH | Brachiation, vertical climbing, leaping, bipedal walking | Sitting, suspensory |
| *Trachypithecus* (*n* = 3)* |  | *Trachypithecus francoisi* (*n* = 1)  *Trachypithecus obscura* (*n* = 1)  *Trachypithecus* sp. (*n* = 1) | M (*n* = 2)  ? (*n* = 1) | EHUB UA | EHUB UA | Arboreal quadrupedal walking, leaping, climbing | Sitting, suspensory, standing |
| Extant platyrrhines | |  |  |  |  |  |  |
| *Alouatta* (*n* = 5) |  | *Alouatta seniculus* (*n* = 3)  *Alouatta seniculus* (*n* = 2) | F (*n* = 3) M (*n* = 2) | AMNH AMNH AMNH | SMiF  AMNH  CASHP | Arboreal quadrupedalism, clambering, suspensory movement, bridging | Sitting, orthograde suspension, lying |
| *Ateles* (*n* = 4) |  | *Ateles fusciceps* (*n* = 4) | F (*n* = 1) | AMNH AMNH AMNH | SMiF  AMNH  CASHP | Suspensory locomotion (brachiation, tail-assisted), quadrupedalism, leaping, clambering | Orthograde suspension, sitting, tail-hangng, lying |

**Table S2.** Individual atlas osteological dimensions (in mm and mm2) of the comparative material. AATh: anterior arch thickness; ALF: area of the superior left articular facet; ARF: area of the superior right articular facet; MDvD: maximum dorsoventral transverse diameter; MTrD: maximum transverse diameter; M10: canal dorsoventral maximum diameter; M11: canal transverse maximum diameter; PaTh: posterior arch thickness; s.d.: standard deviation; STrD: superior transverse diameter; 1L: diameter in major axis of the superior left articular facet; 2L: diameter at a right angle to 1L of the superior left articular facet; 1R: diameter in major axis of the superior right articular facet; 2R: diameter at a right angle to 1R of the superior right articular facet; 1L/2L and 1R/2R: ratio between the diameter in the major axis and the orthogonal diameter. Human individuals are anonymized.

| Taxa | | | Specimens ID | AATh | | MDvD | MTrD | M10 | M11 | PATh | STrD | 1L | 2L | 1L/  2L | ALF | 1R | 2R | 1R/  2R | ARF |
| --- | --- | --- | --- | --- | --- | --- | --- | --- | --- | --- | --- | --- | --- | --- | --- | --- | --- | --- | --- |
| Extant catarrhines | | | |  | |  |  |  |  |  |  |  |  |  |  |  |  |  |  |
| *Cercocebus galeritus* | | 7115 | | 3.6 | | 20.5 | 39.7 | 14.8 | 15.1 | 2.3 | 25.0 | 12.0 | 6.2 | 1.9 | 75.7 | 13.5 | 6.0 | 2.3 | 75.3 |
| *Cercocebus* sp. | | 162 | | 3.3 | | 16.4 | 30.5 | 12.3 | 12.8 | 1.3 | 20.2 | 9.0 | 3.8 | 2.4 | 35.2 | 9.3 | 4.2 | 2.2 | 40.3 |
|  | | mean | | 3.4 | | 18.4 | 35.1 | 13.5 | 13.9 | 1.8 | 22.6 | 10.5 | 5.0 | 2.2 | 55.5 | 11.4 | 5.1 | 2.2 | 57.8 |
|  | | min. | | 3.3 | | 16.4 | 30.5 | 12.3 | 12.8 | 1.3 | 20.2 | 9.0 | 3.8 | 1.9 | 35.2 | 9.3 | 4.2 | 2.2 | 40.3 |
|  | | max. | | 3.6 | | 20.5 | 39.7 | 14.8 | 15.1 | 2.3 | 25.0 | 12.0 | 6.2 | 2.4 | 75.7 | 13.5 | 6.0 | 2.3 | 75.3 |
|  | | s.d. | | 0.2 | | 2.9 | 6.5 | 1.8 | 1.6 | 0.7 | 3.4 | 2.1 | 1.8 | 0.3 | 28.6 | 3.0 | 1.3 | 0.0 | 24.7 |
| *Cercopithecus diana* | | 12403 | | 2.5 | | 18.3 | 36.0 | 14.4 | 13.3 | 1.6 | 21.1 | 10.7 | 5.0 | 2.1 | 47.8 | 10.1 | 5.0 | 2.0 | 46.7 |
| *Cercopithecus diana* | | 12404 | | 2.5 | | 15.9 | 32.2 | 12.3 | 11.6 | 1.3 | 21.3 | 10.4 | 5.2 | 2.0 | 43.4 | 9.6 | 4.4 | 2.2 | 41.8 |
| *Cercopithecus neglectus* | | 7122 | | 3.1 | | 17.1 | 36.9 | 12.7 | 12.9 | 1.4 | 21.9 | 10.5 | 5.0 | 2.1 | 48.0 | 10.0 | 5.6 | 1.8 | 46.2 |
|  | | mean | | 2.7 | | 17.1 | 35.0 | 13.1 | 12.6 | 1.4 | 21.4 | 10.6 | 5.1 | 2.1 | 46.4 | 9.9 | 5.0 | 2.0 | 44.9 |
|  | | min. | | 2.5 | | 15.9 | 32.2 | 12.3 | 11.6 | 1.3 | 21.1 | 10.4 | 5.0 | 2.0 | 43.4 | 9.6 | 4.4 | 1.8 | 41.8 |
|  | | max. | | 3.1 | | 18.3 | 36.9 | 14.4 | 13.3 | 1.6 | 21.9 | 10.7 | 5.2 | 2.1 | 48.0 | 10.1 | 5.6 | 2.2 | 46.7 |
|  | | s.d. | | 0.3 | | 1.2 | 2.5 | 1.1 | 0.9 | 0.2 | 0.4 | 0.2 | 0.1 | 0.1 | 2.6 | 0.3 | 0.6 | 0.2 | 2.7 |
| *Chlorocebus aethiops* | | 12359 | | 2.1 | | 14.8 | 29.1 | 11.0 | 11.6 | 2.1 | 19.5 | 9.5 | 5.5 | 1.7 | 38.3 | 9.7 | 4.2 | 2.3 | 43.9 |
| *Erythrocebus patas* | | 551 | | 2.9 | | 18.0 | 36.2 | 13.1 | 14.0 | 2.2 | 24.8 | 11.1 | 5.0 | 2.2 | 55.3 | 11.5 | 3.1 | 3.6 | 51.1 |
| *Erythrocebus patas* | | 7145 | | 4.2 | | 19.9 | 43.8 | 13.7 | 15.1 | 2.6 | 23.8 | 13.3 | 6.6 | 2.0 | 65.9 | 11.6 | 5.0 | 2.3 | 69.1 |
|  | | mean | | 3.5 | | 19.0 | 40.0 | 13.4 | 14.5 | 2.4 | 24.3 | 12.2 | 5.8 | 2.1 | 60.6 | 11.5 | 4.1 | 3.0 | 60.1 |
|  | | min. | | 2.9 | | 18.0 | 36.2 | 13.1 | 14.0 | 2.2 | 23.8 | 11.1 | 5.0 | 2.0 | 55.3 | 11.5 | 3.1 | 2.3 | 51.1 |
|  | | max. | | 4.2 | | 19.9 | 43.8 | 13.7 | 15.1 | 2.6 | 24.8 | 13.3 | 6.6 | 2.2 | 65.9 | 11.6 | 5.0 | 3.6 | 69.1 |
|  | | s.d. | | 0.9 | | 1.3 | 5.4 | 0.4 | 0.8 | 0.3 | 0.7 | 1.5 | 1.2 | 0.2 | 7.5 | 0.1 | 1.3 | 0.9 | 12.8 |
| *Gorilla beringei* | | 833 | | 5.7 | | 43.7 | 74.5 | 33.1 | 26.4 | 5.5 | 52.2 | 23.8 | 9.6 | 2.5 | 188.1 | 22.8 | 8.4 | 2.7 | 203.3 |
| *Gorilla beringei* | | 8187 | | 7.0 | | 54.6 | 99.2 | 36.9 | 29.4 | 10.6 | 55.6 | 24.5 | 12.0 | 2.0 | 278.1 | 22.5 | 12.4 | 1.8 | 233.9 |
| *Gorilla beringei* | | 54092 | | 5.8 | | 47.4 | 77.7 | 33.8 | 23.5 | 6.7 | 50.9 | 18.2 | 9.6 | 1.9 | 162.9 | 19.0 | 11.1 | 1.7 | 167.9 |
| *Gorilla beringei* | | 8607 | | 6.6 | | 45.9 | 79.1 | 33.2 | 25.1 | 5.0 | 53.4 | 22.9 | 9.6 | 2.4 | 209.8 | 19.9 | 12.4 | 1.6 | 210.6 |
| *Gorilla beringei* | | 397351 | | 8.6 | | 52.5 | 82.4 | 35.1 | 25.5 | 8.8 | 55.6 | 21.1 | 12.6 | 1.7 | 269.2 | 22.2 | 13.6 | 1.6 | 265.7 |
| *Gorilla beringei* | | 395636 | | 6.9 | | 54.4 | 82.4 | 40.0 | 27.6 | 8.5 | 57.7 | 22.0 | 11.9 | 1.8 | 250.2 | 22.6 | 12.0 | 1.9 | 270.3 |
| *Gorilla beringei* | | 1001 | | 5.7 | | 45.2 | 93.6 | 35.5 | 33.9 | 3.1 | 58.2 | 26.9 | 9.0 | 3.0 | 173.7 | 23.9 | 10.8 | 2.2 | 185.4 |
| *Gorilla gorilla* | | 9291 | | 8.8 | | 45.6 | 84.9 | 32.5 | 30.0 | 2.8 | 54.1 | 26.9 | 8.9 | 3.0 | 191.1 | 25.8 | 9.2 | 2.8 | 197.0 |
| *Gorilla gorilla* | | 17202 | | 6.8 | | 40.0 | 71.5 | 29.4 | 27.3 | 2.2 | 48.3 | 22.4 | 9.4 | 2.4 | 225.7 | 22.8 | 8.0 | 2.9 | 255.1 |
| *Gorilla gorilla* | | YPMMAM014998 | | 10.2 | | 46.0 | 74.1 | 33.5 | 31.1 | 3.2 | 50.5 | 28.4 | 10.5 | 2.7 | 266.1 | 25.9 | 11.4 | 2.3 | 226.6 |
| *Gorilla gorilla* | | 5 | | 7.6 | | 48.1 | 92.2 | 37.8 | 32.1 | 3.2 | 57.9 | 23.3 | 9.9 | 2.3 | 175.6 | 25.0 | 10.2 | 2.5 | 194.0 |
| *Gorilla gorilla* | | 23 | | 9.3 | | 56.6 | 96.2 | 37.9 | 35.8 | 11.1 | 58.6 | 28.1 | 13.1 | 2.2 | 174.1 | 29.5 | 10.4 | 2.8 | 215.0 |
| *Gorilla gorilla* | | 176225 | | 9.8 | | 54.5 | 82.5 | 33.3 | 27.3 | 10.1 | 55.2 | 24.9 | 10.4 | 2.4 | 295.3 | 25.2 | 8.9 | 2.8 | 367.5 |
| *Gorilla gorilla* | | 174722 | | 7.5 | | 44.1 | 78.1 | 28.4 | 25.8 | 9.8 | 48.4 | 22.7 | 8.6 | 2.6 | 264.1 | 23.2 | 9.4 | 2.5 | 221.0 |
| *Gorilla gorilla* | | 167340 | | 7.8 | | 48.1 | 67.6 | 35.9 | 28.8 | 4.9 | 49.8 | 24.6 | 8.5 | 2.9 | 192.5 | 22.8 | 6.4 | 3.6 | 179.9 |
| *Gorilla gorilla* | | 586541 | | 7.2 | | 54.8 | 68.4 | 38.1 | 29.0 | 10.4 | 50.1 | 21.7 | 8.6 | 2.5 | 189.5 | 22.8 | 7.9 | 2.9 | 182.7 |
| *Gorilla gorilla* | | 2767 | | 7.7 | | 52.6 | 88.1 | 41.5 | 34.9 | 3.9 | 61.3 | 25.3 | 10.8 | 2.3 | 254.8 | 26.5 | 11.6 | 2.3 | 286.8 |
| *Gorilla gorilla* | | 202932 | | 9.3 | | 56.2 | 100.5 | 36.3 | 30.3 | 11.4 | 65.2 | 29.3 | 12.5 | 2.3 | 404.3 | 30.6 | 12.6 | 2.4 | 383.7 |
|  | | mean | | 7.7 | | 49.4 | 82.9 | 35.1 | 29.1 | 6.7 | 54.6 | 24.3 | 10.3 | 2.4 | 231.4 | 24.0 | 10.4 | 2.4 | 235.9 |
|  | | min. | | 5.7 | | 40.0 | 67.6 | 28.4 | 23.5 | 2.2 | 48.3 | 18.2 | 8.5 | 1.7 | 162.9 | 19.0 | 6.4 | 1.6 | 167.9 |
|  | | max. | | 10.2 | | 56.6 | 100.5 | 41.5 | 35.8 | 11.4 | 65.2 | 29.3 | 13.1 | 3.0 | 404.3 | 30.6 | 13.6 | 3.6 | 383.7 |
|  | | s.d. | | 1.4 | | 5.1 | 10.2 | 3.4 | 3.5 | 3.3 | 4.6 | 2.9 | 1.5 | 0.4 | 60.6 | 2.9 | 2.0 | 0.5 | 60.9 |
| *Homo sapiens* | | Ind_1 | | 8.1 | | 42.7 | 73.7 | 31.8 | 27.7 | 3.4 | 48.8 | 22.0 | 12.1 | 1.8 | 191.9 | 20.1 | 9.6 | 2.1 | 152.3 |
| *Homo sapiens* | | Ind_2 | | 5.7 | | 43.5 | 72.6 | 29.8 | 26.3 | 8.0 | 48.3 | 23.3 | 11.1 | 2.1 | 214.8 | 22.1 | 10.8 | 2.1 | 212.3 |
| *Homo sapiens* | | Ind_3 | | 5.4 | | 43.0 | 72.0 | 32.2 | 30.0 | 5.6 | 52.7 | 23.7 | 10.1 | 2.3 | 164.1 | 21.4 | 10.3 | 2.1 | 195.6 |
| *Homo sapiens* | | Ind_4 | | 6.7 | | 43.2 | 78.4 | 29.0 | 29.0 | 7.7 | 50.4 | 25.2 | 9.7 | 2.6 | 168.6 | 22.1 | 9.1 | 2.4 | 193.2 |
| *Homo sapiens* | | Ind_5 | | 6.2 | | 42.4 | 73.7 | 29.4 | 26.2 | 7.7 | 49.7 | 22.8 | 13.4 | 1.7 | 201.1 | 21.6 | 11.7 | 1.9 | 181.1 |
| *Homo sapiens* | | Ind_6 | | 5.3 | | 42.8 | 73.8 | 30.5 | 28.6 | 7.6 | 48.5 | 26.3 | 12.2 | 2.2 | 216.7 | 24.9 | 9.6 | 2.6 | 216.7 |
| *Homo sapiens* | | Ind_7 | | 6.3 | | 40.4 | 72.2 | 27.9 | 26.8 | 6.6 | 50.4 | 24.5 | 10.6 | 2.3 | 210.0 | 24.3 | 9.4 | 2.6 | 180.2 |
| *Homo sapiens* | | Ind_8 | | 5.7 | | 44.5 | 73.6 | 30.7 | 27.0 | 8.5 | 47.5 | 20.1 | 10.6 | 1.9 | 149.1 | 21.5 | 10.4 | 2.1 | 172.0 |
| *Homo sapiens* | | Ind_9 | | 4.4 | | 38.2 | 64.6 | 27.4 | 26.1 | 6.4 | 40.6 | 19.4 | 8.5 | 2.3 | 164.7 | 23.6 | 8.3 | 2.9 | 185.8 |
| *Homo sapiens* | | Ind_10 | | 4.5 | | 41.8 | 69.2 | 29.5 | 26.0 | 7.2 | 44.8 | 21.2 | 8.9 | 2.4 | 189.0 | 21.3 | 9.0 | 2.4 | 169.5 |
|  | | mean | | 5.8 | | 42.2 | 72.4 | 29.8 | 27.4 | 6.9 | 48.2 | 22.8 | 10.7 | 2.2 | 187.0 | 22.3 | 9.8 | 2.3 | 185.9 |
|  | | min. | | 4.4 | | 38.2 | 64.6 | 27.4 | 26.0 | 3.4 | 40.6 | 19.4 | 8.5 | 1.7 | 149.1 | 20.1 | 8.3 | 1.9 | 152.3 |
|  | | max. | | 8.1 | | 44.5 | 78.4 | 32.2 | 30.0 | 8.5 | 52.7 | 26.3 | 13.4 | 2.6 | 216.7 | 24.9 | 11.7 | 2.9 | 216.7 |
|  | | s.d. | | 1.1 | | 1.8 | 3.6 | 1.5 | 1.4 | 1.5 | 3.4 | 2.2 | 1.5 | 0.3 | 24.1 | 1.5 | 1.0 | 0.3 | 19.6 |
| *Hoolock hoolock* | | 83425 | | 1.7 | | 17.8 | 34.8 | 15.4 | 14.6 | 0.9 | 25.3 | 10.1 | 4.3 | 2.4 | 40.1 | 9.5 | 4.3 | 2.2 | 38.7 |
| *Hylobates agilis* | | 9263 | | 3.6 | | 20.0 | 37.8 | 14.3 | 14.1 | 1.5 | 24.6 | 10.3 | 4.8 | 2.2 | 49.5 | 10.9 | 4.9 | 2.2 | 53.1 |
| *Hylobates agilis* | | 106575 | | 1.9 | | 17.0 | 28.5 | 13.7 | 14.5 | 1.4 | 21.6 | 9.1 | 2.9 | 3.1 | 32.9 | 8.9 | 3.6 | 2.5 | 36.3 |
| *Hylobates klossii* | | 103347 | | 2.7 | | 17.0 | 31.6 | 13.1 | 14.3 | 1.3 | 23.0 | 9.2 | 2.9 | 3.1 | 39.9 | 10.8 | 3.2 | 3.4 | 38.8 |
| *Hylobates lar* | | 9458 | | 4.0 | | 22.9 | 40.3 | 14.8 | 13.5 | 4.1 | 27.8 | 11.0 | 5.3 | 2.1 | 93.6 | 11.3 | 5.6 | 2.0 | 98.6 |
| *Hylobates lar* | | 260590 | | 2.2 | | 17.9 | 33.0 | 14.6 | 14.8 | 1.0 | 24.6 | 10.9 | 4.2 | 2.6 | 40.3 | 11.5 | 4.3 | 2.7 | 49.0 |
| *Hylobates sp* | | DUEA165 | | 2.1 | | 18.4 | 35.3 | 15.2 | 15.5 | 1.4 | 27.0 | 11.4 | 5.4 | 2.1 | 64.4 | 11.2 | 5.0 | 2.2 | 57.0 |
| *Hylobates sp* | | M13956 | | 3.4 | | 32.3 | 60.7 | 25.3 | 21.5 | 3.5 | 34.8 | 15.1 | 4.8 | 3.2 | 106.9 | 16.3 | 5.9 | 2.7 | 118.1 |
| *Hylobates sp* | | 165 | | 2.1 | | 18.3 | 34.9 | 15.2 | 15.5 | 1.2 | 25.8 | 11.8 | 4.1 | 2.9 | 57.0 | 11.4 | 4.2 | 2.7 | 55.0 |
|  | | mean | | 2.8 | | 20.5 | 37.8 | 15.8 | 15.5 | 1.9 | 26.1 | 11.1 | 4.3 | 2.7 | 60.6 | 11.5 | 4.6 | 2.6 | 63.2 |
|  | | min. | | 1.9 | | 17.0 | 28.5 | 13.1 | 13.5 | 1.0 | 21.6 | 9.1 | 2.9 | 2.1 | 32.9 | 8.9 | 3.2 | 2.0 | 36.3 |
|  | | max. | | 4.0 | | 32.3 | 60.7 | 25.3 | 21.5 | 4.1 | 34.8 | 15.1 | 5.4 | 3.2 | 106.9 | 16.3 | 5.9 | 3.4 | 118.1 |
|  | | s.d. | | 0.8 | | 5.2 | 9.9 | 3.9 | 2.5 | 1.2 | 4.0 | 1.9 | 1.0 | 0.5 | 26.7 | 2.1 | 0.9 | 0.4 | 29.2 |
| *Macaca arctoides* | | 112727 | | 3.4 | | 18.1 | 30.9 | 13.6 | 13.6 | 1.4 | 22.9 | 10.4 | 4.4 | 2.4 | 35.7 | 11.2 | 3.5 | 3.2 | 38.7 |
| *Macaca fascicularis* | | 271168 | | 2.9 | | 17.0 | 31.2 | 11.9 | 12.9 | 2.3 | 21.4 | 9.1 | 3.6 | 2.6 | 35.1 | 9.4 | 2.8 | 3.3 | 31.7 |
| *Macaca fuscata* | | 6922 | | 4.7 | | 22.0 | 40.8 | 16.6 | 16.3 | 1.6 | 28.0 | 14.6 | 4.8 | 3.0 | 75.7 | 13.9 | 5.3 | 2.6 | 70.7 |
| *Macaca fuscata* | | 10511 | | 6.1 | | 20.6 | 33.9 | 13.6 | 15.0 | 1.2 | 24.1 | 11.3 | 4.2 | 2.7 | 63.7 | 12.3 | 4.6 | 2.7 | 56.3 |
| *Macaca maura* | | 11982 | | 8.6 | | 24.4 | 38.4 | 14.7 | 14.9 | 2.5 | 24.7 | 11.6 | 5.0 | 2.3 | 78.0 | 11.6 | 4.9 | 2.4 | 65.6 |
| *Macaca mulatta* | | 537253 | | 3.0 | | 19.2 | 36.4 | 14.6 | 13.4 | 1.7 | 23.9 | 11.8 | 4.4 | 2.7 | 57.1 | 11.0 | 4.4 | 2.5 | 52.8 |
| *Macaca mulatta* | | 537241 | | 3.0 | | 16.8 | 32.0 | 12.8 | 14.1 | 1.2 | 21.5 | 9.9 | 3.9 | 2.5 | 40.1 | 9.3 | 4.0 | 2.3 | 38.6 |
|  | | mean | | 4.5 | | 19.7 | 34.8 | 14.0 | 14.3 | 1.7 | 23.8 | 11.3 | 4.3 | 2.6 | 55.1 | 11.2 | 4.2 | 2.7 | 50.6 |
|  | | min. | | 2.9 | | 16.8 | 30.9 | 11.9 | 12.9 | 1.2 | 21.4 | 9.1 | 3.6 | 2.3 | 35.1 | 9.3 | 2.8 | 2.3 | 31.7 |
|  | | max. | | 8.6 | | 24.4 | 40.8 | 16.6 | 16.3 | 2.5 | 28.0 | 14.6 | 5.0 | 3.0 | 78.0 | 13.9 | 5.3 | 3.3 | 70.7 |
|  | | s.d. | | 2.1 | | 2.8 | 3.8 | 1.5 | 1.2 | 0.5 | 2.2 | 1.8 | 0.5 | 0.2 | 18.4 | 1.6 | 0.8 | 0.4 | 14.8 |
| *Nasalis larvatus* | | 4138 | | 5.0 | | 20.9 | 34.1 | 14.8 | 15.1 | 1.5 | 27.4 | 12.8 | 5.2 | 2.5 | 72.9 | 11.9 | 3.8 | 3.1 | 68.2 |
| *Nasalis larvatus* | | 536050 | | 3.3 | | 20.3 | 34.9 | 16.0 | 14.6 | 1.1 | 23.9 | 11.0 | 4.2 | 2.6 | 49.7 | 11.6 | 3.2 | 3.6 | 48.5 |
| *Nasalis larvatus* | | 198276 | | 5.6 | | 22.6 | 42.6 | 16.0 | 16.8 | 1.3 | 28.8 | 12.0 | 5.5 | 2.2 | 75.5 | 11.8 | 4.5 | 2.6 | 62.0 |
|  | | mean | | 4.6 | | 21.2 | 37.2 | 15.6 | 15.5 | 1.3 | 26.7 | 11.9 | 4.9 | 2.4 | 66.0 | 11.7 | 3.8 | 3.1 | 59.6 |
|  | | min. | | 3.3 | | 20.3 | 34.1 | 14.8 | 14.6 | 1.1 | 23.9 | 11.0 | 4.2 | 2.2 | 49.7 | 11.6 | 3.2 | 2.6 | 48.5 |
|  | | max. | | 5.6 | | 22.6 | 42.6 | 16.0 | 16.8 | 1.5 | 28.8 | 12.8 | 5.5 | 2.6 | 75.5 | 11.9 | 4.5 | 3.6 | 68.2 |
|  | | s.d. | | 1.2 | | 1.2 | 4.7 | 0.7 | 1.2 | 0.2 | 2.5 | 0.9 | 0.7 | 0.2 | 14.2 | 0.2 | 0.7 | 0.5 | 10.1 |
| *Pan paniscus* | | 23509 | | 3.7 | | 27.9 | 52.3 | 22.0 | 22.1 | 2.5 | 36.5 | 15.2 | 6.7 | 2.3 | 214.3 | 15.2 | 7.2 | 2.1 | 194.5 |
| *Pan paniscus* | | 29035 | | 3.8 | | 30.1 | 53.0 | 24.3 | 24.5 | 2.1 | 40.7 | 15.9 | 6.9 | 2.3 | 171.5 | 14.1 | 6.9 | 2.0 | 200.7 |
| *Pan paniscus* | | M20191 | | 3.4 | | 32.1 | 60.5 | 25.3 | 21.2 | 3.4 | 43.5 | 14.7 | 7.2 | 2.0 | 144.9 | 15.6 | 7.4 | 2.1 | 154.2 |
| *Pan troglodytes* | | 5891 | | 3.2 | | 32.7 | 60.9 | 24.6 | 24.6 | 4.9 | 44.3 | 20.3 | 10.1 | 2.0 | 222.6 | 18.1 | 11.5 | 1.6 | 261.6 |
| *Pan troglodytes* | | 329 | | 6.0 | | 36.7 | 61.5 | 25.2 | 22.0 | 5.1 | 44.0 | 19.4 | 9.2 | 2.1 | 248.3 | 19.1 | 8.5 | 2.2 | 254.6 |
| *Pan troglodytes* | | Mari | | 4.9 | | 31.9 | 64.6 | 23.0 | 24.4 | 3.4 | 45.5 | 20.1 | 6.9 | 2.9 | 232.9 | 20.4 | 7.0 | 2.9 | 266.2 |
| *Pan troglodytes* | | 9473 | | 5.9 | | 37.4 | 69.5 | 25.5 | 22.0 | 5.0 | 46.9 | 20.7 | 8.4 | 2.5 | 254.5 | 20.3 | 7.0 | 2.9 | 340.6 |
| *Pan troglodytes* | | 8533 | | 4.9 | | 38.2 | 62.6 | 27.6 | 25.0 | 3.6 | 49.6 | 18.3 | 6.7 | 2.7 | 160.1 | 18.5 | 6.9 | 2.7 | 201.1 |
| *Pan troglodytes* | | 184 | | 6.9 | | 35.0 | 67.7 | 24.4 | 23.5 | 5.5 | 49.0 | 16.4 | 7.9 | 2.1 | 222.7 | 17.3 | 8.0 | 2.2 | 203.5 |
| *Pan troglodytes* | | 10814 | | 4.7 | | 35.1 | 59.9 | 25.9 | 24.4 | 3.7 | 42.8 | 15.8 | 6.6 | 2.4 | 279.1 | 18.6 | 7.6 | 2.4 | 266.9 |
| *Pan troglodytes* | | Reiko | | 5.9 | | 39.7 | 66.1 | 26.1 | 23.1 | 7.0 | 45.5 | 20.2 | 6.7 | 3.0 | 237.6 | 26.6 | 7.2 | 3.7 | 249.3 |
| *Pan troglodytes* | | 9266 | | 6.0 | | 34.8 | 67.4 | 25.6 | 25.1 | 3.5 | 47.4 | 19.3 | 6.9 | 2.8 | 182.9 | 22.1 | 6.4 | 3.5 | 198.9 |
| *Pan troglodytes* | | 9803 | | 4.3 | | 35.5 | 64.4 | 24.3 | 20.8 | 7.4 | 39.0 | 17.5 | 9.8 | 1.8 | 146.5 | 17.3 | 7.3 | 2.4 | 163.1 |
| *Pan troglodytes* | | 38 | | 5.9 | | 33.3 | 68.1 | 23.2 | 27.8 | 4.5 | 48.4 | 21.2 | 9.2 | 2.3 | 247.6 | 20.1 | 8.5 | 2.4 | 253.1 |
| *Pan* sp. | | M201606 | | 3.5 | | 29.8 | 45.2 | 23.0 | 21.6 | 3.0 | 37.9 | 16.6 | 6.4 | 2.6 | 221.1 | 15.4 | 7.1 | 2.2 | 223.4 |
|  | | mean | | 4.9 | | 34.0 | 61.6 | 24.7 | 23.5 | 4.3 | 44.1 | 18.1 | 7.7 | 2.4 | 212.4 | 18.6 | 7.6 | 2.5 | 228.8 |
|  | | min. | | 3.2 | | 27.9 | 45.2 | 22.0 | 20.8 | 2.1 | 36.5 | 14.7 | 6.4 | 1.8 | 144.9 | 14.1 | 6.4 | 1.6 | 154.2 |
|  | | max. | | 6.9 | | 39.7 | 69.5 | 27.6 | 27.8 | 7.4 | 49.6 | 21.2 | 10.1 | 3.0 | 279.1 | 26.6 | 11.5 | 3.7 | 340.6 |
|  | | s.d. | | 1.2 | | 3.3 | 6.8 | 1.5 | 1.9 | 1.5 | 4.1 | 2.2 | 1.3 | 0.4 | 41.6 | 3.2 | 1.2 | 0.6 | 48.0 |
| *Papio anubis* | | 1285 | | 5.4 | | 37.5 | 74.3 | 28.0 | 27.4 | 4.6 | 43.1 | 24.7 | 12.7 | 1.9 | 296.8 | 23.8 | 12.2 | 2.0 | 286.3 |
| *Papio anubis* | | 2851 | | 4.0 | | 21.7 | 47.2 | 16.2 | 17.1 | 2.2 | 29.3 | 16.3 | 6.5 | 2.5 | 114.0 | 16.5 | 6.8 | 2.4 | 127.8 |
| *Papio anubis* | | 3250 | | 4.9 | | 25.8 | 54.6 | 16.9 | 16.9 | 3.9 | 30.2 | 17.2 | 6.9 | 2.5 | 154.4 | 17.1 | 7.3 | 2.3 | 153.6 |
| *Papio anubis* | | 384235 | | 3.4 | | 21.6 | 43.5 | 17.1 | 18.3 | 1.6 | 29.5 | 13.5 | 3.8 | 3.5 | 80.9 | 15.1 | 4.0 | 3.8 | 95.5 |
| *Papio anubis* | | 384229 | | 4.4 | | 24.6 | 54.4 | 19.6 | 19.2 | 1.8 | 32.8 | 17.3 | 6.9 | 2.5 | 129.4 | 16.2 | 6.2 | 2.6 | 112.9 |
| *Papio cynocephalus* | | 35413 | | 8.4 | | 38.1 | 69.2 | 27.8 | 28.3 | 3.6 | 43.0 | 21.6 | 9.9 | 2.2 | 210.1 | 24.8 | 7.4 | 3.4 | 208.3 |
| *Papio hamadryas* | | 8949M | | 5.5 | | 35.9 | 63.8 | 26.8 | 26.2 | 4.2 | 46.3 | 23.5 | 9.6 | 2.5 | 319.1 | 24.4 | 9.3 | 2.6 | 304.1 |
| *Papio hamadryas* | | 120388 | | 5.5 | | 28.1 | 57.3 | 18.8 | 18.7 | 4.1 | 33.5 | 15.3 | 7.2 | 2.1 | 115.8 | 14.7 | 7.3 | 2.0 | 132.9 |
| *Papio hamadryas* | | 52668 | | 5.5 | | 28.2 | 46.2 | 19.0 | 19.2 | 3.7 | 31.3 | 17.1 | 5.9 | 2.9 | 87.9 | 17.0 | 6.5 | 2.6 | 109.4 |
|  | | mean | | 5.2 | | 29.0 | 56.7 | 21.1 | 21.2 | 3.3 | 35.4 | 18.5 | 7.7 | 2.5 | 167.6 | 18.9 | 7.4 | 2.6 | 170.1 |
|  | | min. | | 3.4 | | 21.6 | 43.5 | 16.2 | 16.9 | 1.6 | 29.3 | 13.5 | 3.8 | 1.9 | 80.9 | 14.7 | 4.0 | 2.0 | 95.5 |
|  | | max. | | 8.4 | | 38.1 | 74.3 | 28.0 | 28.3 | 4.6 | 46.3 | 24.7 | 12.7 | 3.5 | 319.1 | 24.8 | 12.2 | 3.8 | 304.1 |
|  | | s.d. | | 1.4 | | 6.5 | 10.6 | 4.9 | 4.6 | 1.1 | 6.7 | 3.8 | 2.6 | 0.5 | 88.4 | 4.2 | 2.3 | 0.6 | 78.2 |
| *Pongo abelii* | | 143588 | | 6.5 | | 44.2 | 66.2 | 30.0 | 31.2 | 6.6 | 52.9 | 22.8 | 7.7 | 3.0 | 249.2 | 23.3 | 7.5 | 3.1 | 262.4 |
| *Pongo abelii* | | 150 | | 4.9 | | 33.4 | 58.2 | 26.4 | 23.6 | 2.2 | 46.9 | 19.9 | 7.6 | 2.6 | 175.1 | 20.1 | 7.8 | 2.6 | 175.5 |
| *Pongo pygmaeus* | | 37 | | 5.7 | | 38.2 | 61.4 | 28.8 | 22.9 | 3.1 | 53.7 | 23.2 | 11.4 | 2.0 | 329.3 | 21.3 | 10.1 | 2.1 | 357.5 |
| *Pongo pygmaeus* | | 153823 | | 6.0 | | 38.5 | 48.3 | 28.5 | 22.0 | 4.0 | 48.0 | 22.2 | 9.8 | 2.3 | 275.9 | 22.7 | 11.5 | 2.0 | 252.6 |
| *Pongo pygmaeus* | | 145301 | | 5.9 | | 40.7 | 74.0 | 31.0 | 27.6 | 4.6 | 47.2 | 23.8 | 11.2 | 2.1 | 240.9 | 24.8 | 10.1 | 2.5 | 303.7 |
| *Pongo pygmaeus* | | 145305 | | 6.4 | | 41.1 | 68.2 | 30.1 | 25.4 | 4.1 | 52.0 | 22.0 | 8.8 | 2.5 | 224.0 | 23.2 | 10.7 | 2.2 | 225.1 |
| *Pongo pygmaeus* | | 145302 | | 4.1 | | 32.4 | 62.2 | 27.0 | 23.0 | 1.4 | 40.7 | 18.4 | 8.3 | 2.2 | 192.0 | 20.9 | 9.3 | 2.3 | 247.0 |
| *Pongo pygmaeus* | | 153805 | | 4.9 | | 33.8 | 48.9 | 26.2 | 19.9 | 3.0 | 40.5 | 18.5 | 8.0 | 2.3 | 153.2 | 20.4 | 6.7 | 3.0 | 155.7 |
| *Pongo pygmaeus* | | 588109 | | 4.8 | | 38.0 | 59.5 | 29.6 | 21.2 | 4.6 | 60.2 | 21.5 | 10.0 | 2.1 | 339.1 | 22.8 | 9.9 | 2.3 | 293.6 |
| *Pongo pygmaeus* | | 200900 | | 3.5 | | 34.4 | 57.9 | 28.4 | 23.1 | 4.4 | 41.2 | 19.3 | 7.4 | 2.6 | 152.3 | 18.0 | 7.3 | 2.5 | 145.0 |
|  | | mean | | 5.3 | | 37.5 | 60.5 | 28.6 | 24.0 | 3.8 | 48.3 | 21.2 | 9.0 | 2.4 | 233.1 | 21.7 | 9.1 | 2.4 | 241.8 |
|  | | min. | | 3.5 | | 32.4 | 48.3 | 26.2 | 19.9 | 1.4 | 40.5 | 18.4 | 7.4 | 2.0 | 152.3 | 18.0 | 6.7 | 2.0 | 145.0 |
|  | | max. | | 6.5 | | 44.2 | 74.0 | 31.0 | 31.2 | 6.6 | 60.2 | 23.8 | 11.4 | 3.0 | 339.1 | 24.8 | 11.5 | 3.1 | 357.5 |
|  | | s.d. | | 1.0 | | 3.9 | 8.0 | 1.6 | 3.3 | 1.5 | 6.5 | 2.0 | 1.5 | 0.3 | 67.2 | 2.0 | 1.6 | 0.4 | 68.3 |
| *Pygathrix nemaeus* | | 12438 | | 3.9 | | 20.6 | 34.5 | 14.7 | 14.6 | 2.3 | 24.9 | 12.1 | 4.1 | 3.0 | 54.4 | 12.0 | 5.0 | 2.4 | 58.7 |
| *Semnopithecus entellus* | | 2942 | | 4.0 | | 19.3 | 38.0 | 14.0 | 13.6 | 1.7 | 24.4 | 10.2 | 3.3 | 3.1 | 42.8 | 10.5 | 3.6 | 2.9 | 51.7 |
| *Symphalangus syndactylus* | | 106581 | | 3.0 | | 22.5 | 38.8 | 17.1 | 16.3 | 2.3 | 28.5 | 13.0 | 4.5 | 2.9 | 54.4 | 12.8 | 3.9 | 3.3 | 53.2 |
| *Trachypithecus obscura* | | 12131 | | 4.3 | | 18.5 | 33.0 | 12.5 | 13.6 | 2.1 | 24.5 | 11.4 | 4.6 | 2.5 | 60.6 | 11.8 | 5.8 | 2.0 | 70.9 |
| *Trachypithecus francoisi* | | 12436 | | 3.7 | | 18.5 | 34.5 | 12.7 | 14.4 | 2.0 | 23.4 | 11.5 | 5.3 | 2.2 | 52.7 | 10.9 | 6.6 | 1.7 | 54.5 |
| *Trachypithecus* sp. | | 161 | | 2.0 | | 14.6 | 24.6 | 12.0 | 11.0 | 1.1 | 18.2 | 8.8 | 3.4 | 2.6 | 27.9 | 8.7 | 4.1 | 2.1 | 29.0 |
|  | mean | | | | 4.0 | 17.8 | 32.4 | 12.3 | 13.3 | 1.8 | 24.0 | 10.7 | 3.8 | 3.0 | 51.9 | 10.2 | 3.4 | 3.1 | 45.1 |
|  | min. | | | | 3.3 | 15.8 | 27.0 | 11.4 | 12.1 | 1.0 | 19.6 | 9.7 | 2.3 | 2.1 | 31.5 | 9.2 | 2.9 | 2.5 | 31.7 |
|  | max. | | | | 4.7 | 19.0 | 40.5 | 13.1 | 14.4 | 2.9 | 26.2 | 11.9 | 4.9 | 4.5 | 65.4 | 11.8 | 3.8 | 3.5 | 62.6 |
|  | s.d. | | | | 0.6 | 1.3 | 6.2 | 0.7 | 1.1 | 0.8 | 2.6 | 0.8 | 1.0 | 1.0 | 12.5 | 1.1 | 0.4 | 0.4 | 11.9 |
| *Extant platyrrhines* | | | |  | |  |  |  |  |  |  |  |  |  |  |  |  |  |  |
| *Alouatta seniculus* | | 200550 | | 4.3 | | 17.1 | 27.8 | 11.4 | 12.3 | 1.6 | 24.1 | 9.7 | 4.3 | 2.2 | 54.2 | 9.2 | 3.7 | 2.5 | 47.0 |
| *Alouatta seniculus* | | 42316 | | 3.6 | | 18.6 | 29.1 | 13.1 | 13.1 | 2.5 | 24.1 | 10.4 | 4.9 | 2.1 | 56.4 | 11.8 | 3.4 | 3.5 | 47.6 |
| *Alouatta seniculus* | | 23333 | | 4.0 | | 18.4 | 37.7 | 12.8 | 14.4 | 1.4 | 26.0 | 11.2 | 3.5 | 3.2 | 65.4 | 11.1 | 3.8 | 2.9 | 62.6 |
| *Alouatta palliata* | | 282798 | | 3.3 | | 15.8 | 27.0 | 12.0 | 12.1 | 1.0 | 19.6 | 10.4 | 2.3 | 4.5 | 31.5 | 9.6 | 3.1 | 3.1 | 31.7 |
| *Alouatta palliata* | | 338107 | | 4.7 | | 19.0 | 40.5 | 12.0 | 14.4 | 2.9 | 26.2 | 11.9 | 3.9 | 3.0 | 51.9 | 9.5 | 2.9 | 3.3 | 36.7 |
|  | | mean | | 4.0 | | 17.8 | 32.4 | 12.3 | 13.3 | 1.8 | 24.0 | 10.7 | 3.8 | 3.0 | 51.9 | 10.2 | 3.4 | 3.1 | 45.1 |
|  | | min. | | 3.3 | | 15.8 | 27.0 | 11.4 | 12.1 | 1.0 | 19.6 | 9.7 | 2.3 | 2.1 | 31.5 | 9.2 | 2.9 | 2.5 | 31.7 |
|  | | max. | | 4.7 | | 19.0 | 40.5 | 13.1 | 14.4 | 2.9 | 26.2 | 11.9 | 4.9 | 4.5 | 65.4 | 11.8 | 3.8 | 3.5 | 62.6 |
|  | | s.d. | | 0.6 | | 1.3 | 6.2 | 0.7 | 1.1 | 0.8 | 2.6 | 0.8 | 1.0 | 1.0 | 12.5 | 1.1 | 0.4 | 0.4 | 11.9 |
| *Ateles fusciceps* | | 188141 | | 2.2 | | 18.7 | 35.9 | 14.4 | 12.9 | 2.3 | 26.1 | 10.6 | 4.9 | 2.2 | 60.7 | 9.9 | 4.6 | 2.1 | 54.2 |
| *Ateles fusciceps* | | 188135 | | 2.9 | | 17.2 | 35.1 | 12.4 | 13.9 | 2.6 | 24.8 | 10.6 | 4.2 | 2.5 | 61.8 | 11.3 | 4.1 | 2.8 | 62.6 |
| *Ateles fusciceps* | | 188140 | | 2.4 | | 19.7 | 38.9 | 15.9 | 15.9 | 1.6 | 27.5 | 10.1 | 5.3 | 1.9 | 50.6 | 11.6 | 5.0 | 2.3 | 57.8 |
| *Ateles fusciceps* | | 338112 | | 2.5 | | 19.8 | 38.5 | 15.5 | 15.2 | 2.1 | 28.4 | 12.6 | 3.9 | 3.2 | 41.7 | 12.6 | 3.7 | 3.4 | 49.0 |
|  | | mean | | 2.5 | | 18.8 | 37.1 | 14.6 | 14.5 | 2.1 | 26.7 | 11.0 | 4.6 | 2.5 | 53.7 | 11.4 | 4.4 | 2.7 | 55.9 |
|  | | min. | | 2.2 | | 17.2 | 35.1 | 12.4 | 12.9 | 1.6 | 24.8 | 10.1 | 3.9 | 1.9 | 41.7 | 9.9 | 3.7 | 2.1 | 49.0 |
|  | | max. | | 2.9 | | 19.8 | 38.9 | 15.9 | 15.9 | 2.6 | 28.4 | 12.6 | 5.3 | 3.2 | 61.8 | 12.6 | 5.0 | 3.4 | 62.6 |
|  | | s.d. | | 0.3 | | 1.2 | 1.9 | 1.6 | 1.3 | 0.4 | 1.6 | 1.1 | 0.6 | 0.6 | 9.5 | 1.1 | 0.6 | 0.6 | 5.7 |

**Table S3.** Procrustes distances between *Otavipithecus* and all extant group means computed from the Procrustes-registered shape coordinates of the complete atlas morphology.

| Taxa | Procrustes distances |
| --- | --- |
| Extant catarrhines |  |
| *Cercocebus* | 93.9 |
| *Cercopithecus* | 98.3 |
| *Chlorocebus* | 105.7 |
| *Erythrocebus* | 95.4 |
| *Gorilla* | 91.4 |
| *Homo* | 85.3 |
| *Hoolock* | 114.3 |
| *Hylobates* | 87.6 |
| *Macaca* | 92.5 |
| *Nasalis* | 119.5 |
| *Pan* | 79.8 |
| *Papio* | 82.4 |
| *Pongo* | 89.9 |
| *Pygathrix* | 98.8 |
| *Semnopithecus* | 100.4 |
| *Symphalangus* | 113.2 |
| *Trachypithecus* | 87.6 |
| Extant platyrrhines |  |
| *Alouatta* | 128.8 |
| *Ateles* | 109.1 |

**Table S4.** Procrustes distances between *Otavipithecus* and *Nacholapithecus* and all extant group means computed from the Procrustes-registered shape coordinates of the partial atlas morphology.

| Taxa | Procrustes distances | |
| --- | --- | --- |
|  | *Otavipithecus* | *Nacholapithecus* |
| Extant catarrhines |  |  |
| *Cercocebus* | 42.1 | 33.6 |
| *Cercopithecus* | 42.4 | 37.9 |
| *Chlorocebus* | 34.2 | 27.9 |
| *Erythrocebus* | 30.9 | 29.3 |
| *Gorilla* | 29.1 | 32.5 |
| *Homo* | 33.9 | 34.5 |
| *Hoolock* | 36.3 | 34.7 |
| *Hylobates* | 23.7 | 25.6 |
| *Macaca* | 31.6 | 28.1 |
| *Nasalis* | 22.7 | 21.8 |
| *Pan* | 27.4 | 35.2 |
| *Papio* | 24.1 | 25.5 |
| *Pongo* | 31.7 | 32.4 |
| *Pygathrix* | 34.7 | 43.6 |
| *Semnopithecus* | 35.4 | 31.7 |
| *Symphalangus* | 46.1 | 44.7 |
| *Trachypithecus* | 33.6 | 31.6 |
| Extant platyrrhines |  |  |
| *Ateles* | 40.3 | 38.6 |
| *Alouatta* | 47.1 | 45.0 |
|  |  |  |
|  |  |  |


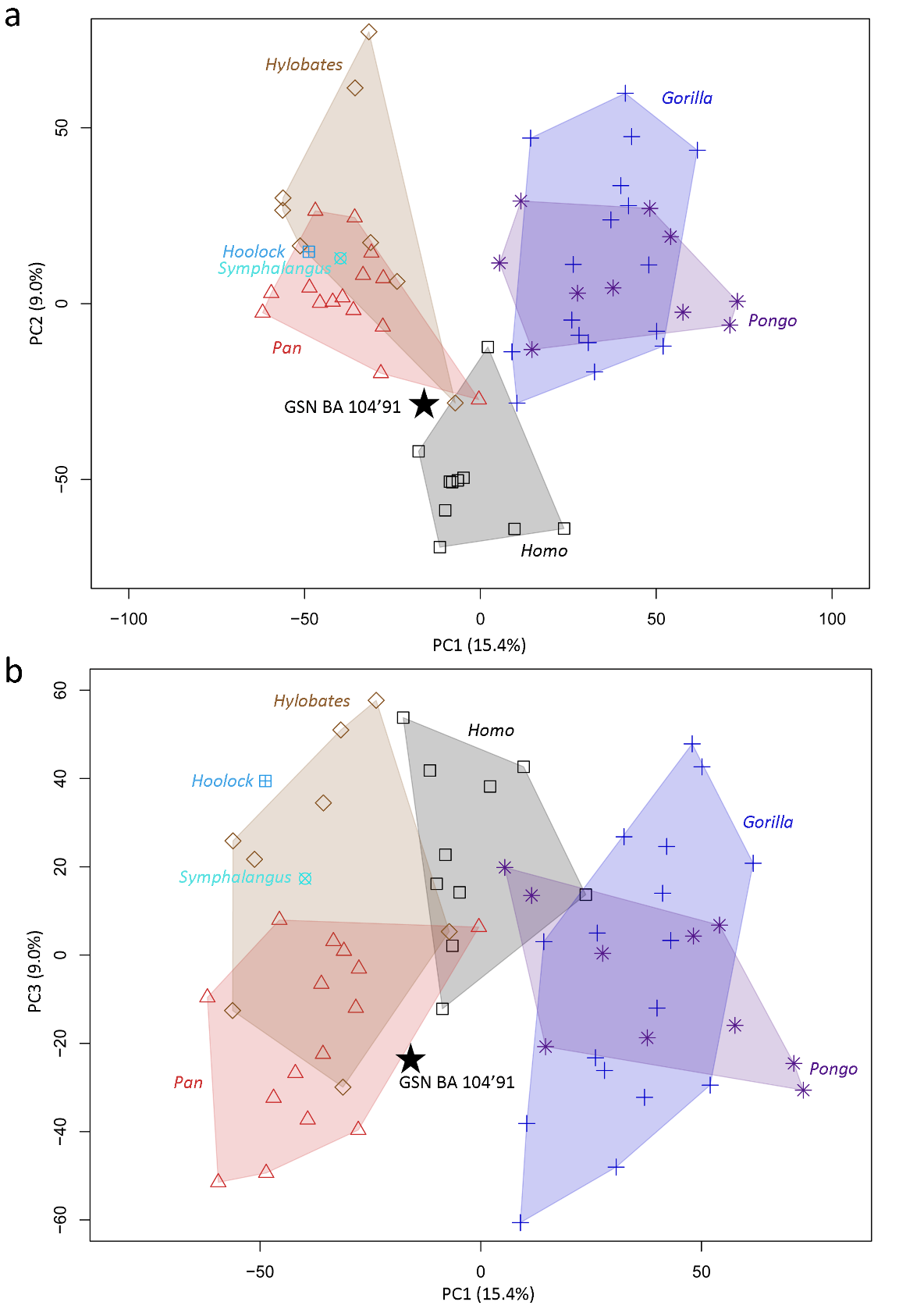


**Figure S1.** Principal component analysis (PCA) of the Procrustes-registered shape coordinates of the complete atlas morphology calculated for GSN BA 104’91 and comparative extant hominoids for PC1, PC2 (**a**) and PC3 (**b**).


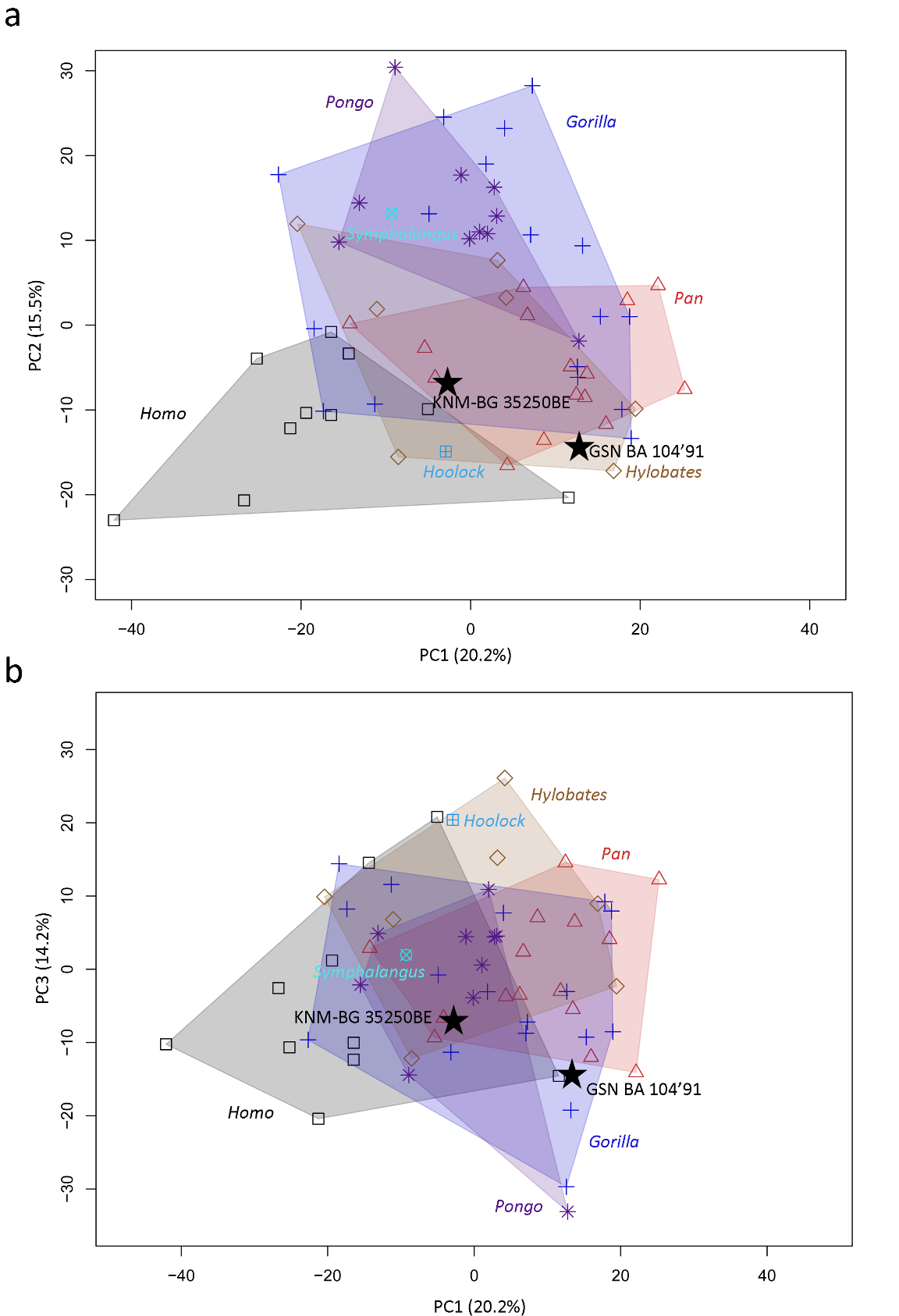


**Figure S2.** Principal component analysis (PCA) of the Procrustes-registered shape coordinates of the partial atlas morphology calculated for GSN BA 104’91 and KNM-BG 35250BE, and comparative extant hominoids for PC1, PC2 (**a**) and PC3 (**b**).

**References**

Gebo, D. L. Climbing, brachiation, and terrestrial quadrupedalism: Historical precursors of hominid bipedalism. *Am. J. Phys. Anthropol.* **101**, 55–92 (1996).

Hunt, K. D. Positional behavior in the Hominoidea. *Int. J. Primatol.* **12**, 95–118 (1991).

Hunt, K. D. *et al.* Standardized descriptions of primate locomotor and postural modes. *Primates* **37**, 363–387 (1996).

Estrada, G. R. & Marshall, A. J. Terrestriality across the primate order: A review and analysis of ground use in primates. *Evol. Anthropol. Issues News Rev.* **33**, e22032 (2024).
